# Primary cilia in growth plates orchestrate long bone development

**DOI:** 10.1016/j.fmre.2025.04.014

**Published:** 2025-05-03

**Authors:** Lei Zhang, Xiaoqiao Xu, Dike Tao, Xinyu Li, Pingping Niu, Xuyan Gong, Gongchen Li, Mengfei Yu, Yao Sun

**Affiliations:** a Shanghai Engineering Research Center of Tooth Restoration and Regeneration & Tongji Research Institute of Stomatology & Department of Implantology, Shanghai Tongji Stomatological Hospital and Dental School, Tongji University, Shanghai 200072, China; b Shanghai Engineering Research Center of Tooth Restoration and Regeneration & Tongji Research Institute of Stomatology & Department of Oral and Maxillofacial Surgery, Shanghai Tongji Stomatological Hospital and Dental School, Tongji University, Shanghai 200092, China; c Stomatology Hospital, School of Stomatology, Zhejiang University School of Medicine, Zhejiang Provincial Clinical Research Center for Oral Diseases, Key Laboratory of Oral Biomedical Research of Zhejiang Province, Engineering Research Center of Oral Biomaterials and Devices of Zhejiang Province, Zhejiang University, Hangzhou 311399, China

**Keywords:** Primary cilia, Growth plate, Wnt, Long bone development, Skeletal stem cell

## Abstract

The growth plate plays a crucial role in long bone development and elongation, housing stem cells that contribute to bone formation. This study investigates the role of primary cilia, specialized organelles that regulate stem cell fate, in the development and repair of long bones. Here, we report the presence of primary cilia in all zones of the growth plate, particularly during embryonic development. Spatial transcriptomics and analysis of conditional knockout (CKO) mice identified that primary cilia mediate critical developmental signaling pathways within the growth plate. Disruption of primary cilia in growth plate chondroblasts or osteoblasts impaired long bone development by activating the Wnt signaling pathway and disrupting the cellular stemness. This resulted in elevated Mmp13 secretion, abnormal mineralization, and structural defects, ultimately hindering bone elongation. Time-lapse imaging showed an increased frequency of abnormal mitosis and a reduced rate of asymmetric division in skeletal stem cells (SSCs) from CKO mice. In conclusion, our findings indicated that primary cilia are critical for long bone development, regulating stem cell fate through key signaling pathways. Loss of primary cilia leads to excessive Wnt signaling and disruption of SSC stemness, impairing bone elongation. This study highlights the essential role of primary cilia in bone development and suggests potential therapeutic targets for skeletal disorders.

## Graphical abstract

**Figure:**
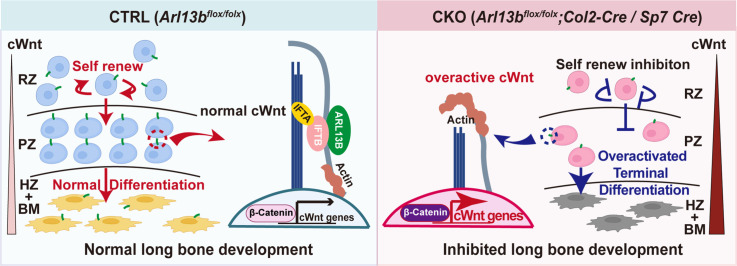

## 1 Introduction

The development of the skeletal system is a significant concern in contemporary society [1], as premature stagnation can severely impair patients' physical and mental health. The development of long bones is primarily mediated by the endochondral ossification process [2,3], which progresses continuously during bone development [4]. This process critically depends on the growth plate [4,5], a cartilaginous structure located at the metaphysis of bones [6]. Abnormal development of the growth plate can lead to disorders of bone formation and hinder bone elongation [7,8]. Numerous studies have demonstrated that various signaling pathways [9], including Wnt [10], TGF-β [11], BMP [12], FGF [13], and mTOR [14], regulate growth plate development, collectively forming a complex regulatory network [6,15,16]. Consequently, precise targeting and modulation of this signaling network may enhance the mediation of long bone development.

Skeletal stem cells (SSCs), a type of mesenchymal stem cells widely distributed throughout different regions of long bones, play a crucial role in mediating bone development and repair [17,18]. During the development, SSCs must balance self-renewal with the generation of differentiating progeny, necessitating that quiescent and metabolically inactive SSCs undergo division to produce daughter cells [19]. The activation of SSCs carries the risk of exhaustion [20,21], which can significantly impair bone development. Conversely, the production of additional SSCs and differentiated progeny requires these cells to exit quiescence and divide, a process that is also critical for bone elongation [22]. However, the mechanisms by which intracellular and extracellular signaling regulates SSC activation and quiescence remain incompletely understood.

Primary cilia are specialized cellular receptors that protrude from the surface of most eukaryotic cells [23] and host receptor molecules for multiple signaling pathways [24,25]. During tissue development and homeostasis, primary cilia harbor signaling molecules that govern cellular activities [26,27], acting as pivotal transmission hubs for diverse signaling pathways [28,29]. Previous studies have indicated that structural abnormalities in primary cilia can adversely affect the repair of growth plate damage [30]. However, their potential role as a signaling hub for the precise mediation of long bone development and growth requires further investigation.

Our research confirms the extensive presence of primary cilia in cells across the growth plate, with heightened activity observed during growth and developmental stages. Conditional knockout of the key ciliary membrane gene Arl13b in growth plate chondroblasts results in structural disruption of the growth plate in mice, impeding growth and development. Further analysis of spatial and transcriptome sequencing data [31] reveals that the regulation of growth plate stem cell fate specification and the Wnt signaling pathway are intimately linked to primary cilia membrane gene expression. Conditional knockout of primary cilia disrupts signaling hub homeostasis, characterized by excessive activation of Wnt signaling and attenuation of Hedgehog (Hh) signaling pathways. Additionally, the time-lapse imaging revealed an increased percentage of abnormal cell mitosis as well as a decreased percentage of asymmetric division among SSCs, indicating that cell fate is mediated by primary cilia. Whereas suppressing Wnt signaling partially reverses the phenotype in these CKO mice. These results underscore the pivotal role of developmental signaling pathways mediated by primary cilia in the growth and development of long bones.

In summary, we systematically analyzed the role of primary cilia within different cells of the long bone growth plate, revealing the influence of long bone development by orchestrating critical developmental signaling pathways and mediating the cell fate specification of chondrocytes within the growth plate. Our study confirms that primary cilia function as signal stabilizers during the growth plate-mediated long bone development process. Structural abnormalities in primary cilia can lead to excessive upregulation of Wnt signaling and increased secretion of MMP13, resulting in structural disorder of the growth plate. Our research further highlights the critical role of primary cilia in the development of long bones.

## 2 Materials and methods

### 2.1 Mice strain

All mice were maintained in a specific pathogen-free (SPF) facility under a 12/12h day/night illumination cycle. They were euthanized by cervical dislocation after inhalation anesthesia. All mice procedures were approved by the Animal Welfare Committee of Tongji University (Protocol numbers: [2022]-DW-06) and followed all ARRIVE recommendations (Animal Studies: Reporting of *In Vivo* Experiments) guidelines.

All mice were maintained on the C57BL/6 background, including Arl13b*^flox/flox^*, Col2a1-Cre and Sp7-Cre. They were housed in SPF facilities of the Animal Resource Center at Tongji University.

### 2.2 Animal experiments

All the samples were specifically restricted to males. The sample size was no less than six animals for all experiments. Specific numbers are stated in the figure legends. The sample size was determined by the number of viable animals of the right age and genotype at the time of the experiment. All data analysis was conducted in a blinded manner for experiments in which the investigator could affect the outcome, such as any µCT analyses, cell counting in immunofluorescence-based assays, assessment of molecular treatments, and so on. Animals with the appropriate genotype were randomly allocated to experimental conditions.

#### 2.2.1 Animal experiments

As for joint injection, DMSO and Wnt inhibitor ICRT3 was injected into the joint cavity of 2-week-old control and CKO mice, respectively. The injection was applied twice on postnatal day 14 and 16 of mice. Subsequently, we analyzed femoral cortical bone and trabecular phenotype using µ-CT after 6 weeks. Animals were allowed to move freely in the cage after surgery with access to food and water.

### 2.3 Micro-CT analysis

Dissected femurs were fixed in 4% paraformaldehyde (PFA) for 48 hours. The femurs were subjected to analysis by micro–CT 50 (Scanco Medical, Zurich, Switzerland) at a scan resolution of a 14-µm slice increment with a voltage of 70 kv and a current of 200µA. 100 slices of trabecular bone underneath the growth plate were reconstructed for the statistical analysis. The parameters of trabecular bone volume/total volume (BV/TV), trabecular number (Tb.N), trabecular thickness (Tb.Th), and trabecular separation (Tb.Sp) were quantified according to the standard procedures.

### 2.4 Histological assessment

For histological analysis, femurs were decalcified in 10% EDTA (pH 7.4) at 4 °C for 2–4 weeks. After being dehydrated through a graded ethanol series, specimens were embedded in paraffin and cut into 5 µm-thick sections. For morphological evaluation, hematoxylin and eosin (H&E) (Sangon Biotech, Shanghai, China) staining was performed. For extracellular matrix analysis in the growth plate, the sections were stained with Safranin O/Fast Green. (Solarbio, Beijing, China) according to the manufacturer’s protocol. Staining of skeletons with Alcian blue and Alizarin red was performed as described [32].

### 2.5 Immunofluorescence and confocal imaging

For immunofluorescence staining, femurs were embedded in OCT and sectioned at an 8-µm thickness after being decalcified. Antigen retrieval was performed using hyaluronidase; sections were then blocked in 5% FBS in PBS-T (0.1% Triton X-100 in PBS). The sections were incubated overnight at 4 °C with anti- type II collagen (COL II) (1:200, Boster Biological Technology, Wuhan, China), anti-γ-tubulin (1:1000, Sigma-Aldrich. St Louis, MO), anti-acetylated α-tubulin (1:1000, Sigma-Aldrich. St Louis, MO), anti-ARL13B (1:200, Proteintech Group, Wuhan, China), anti-Matrix metalloproteinases13 (Mmp13) (1:200, Proteintech Group, Wuhan, China), anti-β-catenin (1:200, Proteintech Group, Wuhan, China), anti-Smo (1:200, Proteintech Group, Wuhan, China), anti-Sp7 (1:200, Abcam, USA), anti-Runx2 (1:200, Abcam, USA), anti-Sox9 (1:200, Abcam, USA), anti-Ihh (1:200, Abcam, USA), anti - endomucin (EMCN) (1:200, Santa Cruz Biotechnology, USA), On the second day, the sections were incubated with the appropriate secondary antibody conjugated with fluorophore (Invitrogen. Carlsbad, California). Sections were subsequently stained with DAPI (Sigma-Aldrich. St Louis, MO). All fluorescence microscopy images were acquired using a Nikon TI2-*E* + A1 R confocal microscope (Nikon, Japan) or Eclipse Ni-U microscope (Nikon, Japan). Imaris Microscopy Image Analysis Software (Oxford instruments) was used for quantitative analysis. The catalog numbers for all the antibodies used are listed in Table S1.

### 2.6 RNA-sequencing

Total RNA was extracted from Control and CKO mice growth plates using Trizol reagent (Invitrogen). RNA integrity was assessed using the RNA Nano 6000 Assay Kit of the Bioanalyzer 2100 system (Agilent Technologies, CA, USA). Total RNA was used as input material for the RNA sample preparations. PCR products were purified (AMPure XP system), and library quality was assessed on the Agilent Bioanalyzer2100 system. The clustering of the index-coded samples was performed on a cBot Cluster Generation System using TruSeq PE Cluster Kit v3-cBot-HS (lllumia). RNA-seq reads were mapped to transcripts from Mus - musculus.

Differential expression analysis of two groups (three biological replicates per condition) was performed using the DESeq2R package (1.20.0). The resulting P-values were adjusted using the Benjamini and Hochberg's approach for controlling the false discovery rate. Genes with an adjusted *P value* < 0.05 found by DESeq2 were assigned as differentially expressed. Gene Ontology (GO) enrichment analysis of differentially expressed genes was implemented by the cluster Profiler R package, in which gene length bias was corrected. GO terms with a corrected *P value* < 0.05 were considered significantly enriched by differentially expressed genes. We used the cluster Profiler R package to test the statistical enrichment of differential expression genes in KEGG pathways.

### 2.7 Re-analysis of RNA-seq data

The Spatial Transcriptome data were obtained from the GEO database (GSE221571) [31]. The analysis was performed using R version 4.3.0. Seurat version 5.1.0. and Cell Chat version 1.6.1. Briefly, the expression matrix was imported to the Seurat (4.3.0.). After quality control and normalization, UMAP was used to visualize the coordinates of all cells and “Find All Markers” was used to calculate differentially expressed genes (DEGs). For stacked violin plots, “VlnPlot” of MySeuratWrappers (R package) was used (“stacked = TRUE”). The mean expression level of genes was calculated and presented by pheatmap (R package) with row-wide z-score. Cell-to-cell interactions were calculated by CellChat (R package).

### 2.8 Cell culture

The primary cells were maintained at 37 °C and 5% CO_2_. DMEM (Gibco) with 2% FBS containing ITS supplement (Sigma I3146) and vitamin C was gently added to induce chondrocyte differentiation.

### 2.9 Scratch-wound assay

Primary skeletal stem cells were plated into a 24-well culture plate and placed at 37  °C, 5% CO_2_ for 24 h. Remove the medium and scratch the surface of the inoculated cells with a 10 µL pipette tip. Wash the cells gently twice with PBS. Photograph the scratches at 0 h, 24h and 72 h, respectively. The distance that the cells migrated to the wounded area during this time was measured by ImageJ software.

### 2.10 EdU cell proliferation assay

EdU (5-ethynyl-2′-deoxyuridine, Beyotime, Shanghai, China) dissolved in PBS was administered to mice once, at six hours before euthanization at the indicated days (500µg for 4w per injection). BeyoClick™ EdU- 488, 594 or 647 (Beyotime, Shanghai, China) was used to detect EdU in paraffin section.

### 2.11 *In vitro* primary cell differentiation

*In vitro* primary growth plate cells were cultured for 7 days and passaged into 24-well plates at a cell density of 3 × 10^4^/cm^2^. After confluence, the cell medium was changed to a chondrogenetic or osteogenic medium. The osteogenic medium contains α-MEM (Gibco, USA), 10% FBS (Gibco, USA), 1 mM sodium pyruvate, 1% P/S, 50 mg/ml l-ascorbic acid, 10 mM β-glycerophosphate, and 100 nM dexamethasone. The osteogenic medium was changed every 3 days. Alkaline phosphatase (ALP) (Beyotime, Shanghai, China) and Alizarin red (Sigma, USA) staining was performed on day 7 and day 14, respectively, according to the manufacturers’ protocols. The primary cells were maintained at 37 °C and 5% CO_2_. DMEM (Gibco, USA) with 2% FBS containing ITS supplement (Sigma, USA) and vitamin C was gently added to induce chondrocyte differentiation. The q-PCR assay was performed on days 14 and 21.

### 2.12 Quantitative real-time PCR (q-PCR) assay

The total RNA was isolated from tissues or cells with RNA iso Plus reagent (Life, Shanghai, China). cDNA was synthesized using a PrimeScript™ RT reagent Kit with gDNA Eraser (Perfect Real Time) (TaKaRa, Beijing, China). q-PCR was conducted with a SYBR Premix Ex Taq II kit (Yeasen, Shanghai, China). The levels of mRNAs were normalized to that of the housekeeping gene Gapdh. All q-PCR procedures, including the design of the primers, validation of PCR conditions and quantification, were performed according to MIQE guidelines. Gene-specific primer sequences are listed in Table S2.

### 2.13 Flow cytometry

The growth plate of femurs was manually separated under the stereo microscope, and soft tissues were attached using forceps. The dissected growth plate was minced using microsurgical scissors and incubated with 3mg/ml collagenase I and 0.2% collagenase II in Hank’s Balanced Salt Solution (HBSS, Sigma-Aldrich. St Louis, MO) at 37 °C for 30 min for twice. Cells were mechanically triturated using a vortex and filtered through a 70 µm cell strainer (Corning, NY, USA) into a 15 ml tube on ice to single cell suspension. After washing, tissue remnants were incubated with collagenase at 37 °C for another 30 min, and cells were filtered into the same tube. Cells were pelleted and resuspended in FACS buffer (Hank’s buffer, 2% FBS, 5mM EDTA) for flow cytometry. Dissociated cells were stained by standard protocols with the following antibodies (eBioscience, Shanghai, China): allophycocyanin (APC)-conjugated CD31 (MEC13.3), CD45 (30F-11), Ter119 (TER-119), Thy1.2(5E10), PE/Cy5-CD105 (MJ7/18), and PE/Cy7-conjugated CD200 (OX-90). DAPI was used for viable cell gating. Flow cytometry analysis was performed using BD FACS AriaIII and BD FACS Diva software. Acquired raw data were further analyzed on FlowJo version 10.9.0. The catalog numbers for the antibodies used are listed in Table S1.

### 2.14 Immunohistochemistry

For immunohistochemical analysis of phosphorylated-βcatenin (p-βcatenin), dissected femurs were first fixed in 4%PFA, before they were decalcified in EDTA and embedded in paraffin. Sections of 5 µm thickness were cut, and after deparaffinization, antigen retrieval was performed using hyaluronidase. Sections were then blocked in 5% FBS in PBS-T (0.1% Triton X-100 in PBS). The sections were incubated overnight at 4 °C with anti-p-β-catenin (1:200, Proteintech Group, Wuhan, China). Sections were treated with streptavidin–horseradish peroxidase (HRP), and peroxidase activity was visualized using the DAB^+^ substrate-chromogen solution. Finally, the slides were counterstained with hematoxylin and mounted. The catalog numbers for all the antibodies used are listed in Table S1.

### 2.15 Time-lapse imaging

The primary cells from both control and CKO groups applied in this experiment were sorted through FACS based on the mSSCs marker (CD200^+^CD105^-^). After the confluence of cells reached 50%, the cells were incubated with Sir-tubulin (1:1000, Spirochrome, Switzerland) or LysoBrite (1:1000, AAT Bioquest, USA) at 37 °C for 1 hour. Then, the cells were washed with PBS to remove the probe dye solution. All the time-lapse imaging was acquired using a Nikon TI2-*E* + A1 R confocal microscope (Nikon, Japan) for 24 hours under 37 °C, 5% CO_2_ cell culture environment.

### 2.16 Genotyping

Mice tail tips were lysed using DirectPCR Lysis Reagent (Viagen Biotech, 102-T) according to the manufacturer’s instructions. PCRs were performed to identify mice with Cre recombinase and/or loxP sites with the following primers: Arl13b-flox: 5′- ATGACCCTGCATAACTCAATACAGA-3′, 5′-GGTAAAGAAACCAAGGAAGGGATGA-3′ (wild type = 160 bp, heterozygote = 223 bp and 160 bp, homozygote = 223 bp); Col2-cre: 5′-GCGGTCTGGCAGTAA AAACTATC-3′, 5′-GTGAAACAGCATTGCTGTCACTT-3′ (wild type = 100 bp, heterozygote = 324 bp and 100 bp, homozygote = 324 bp); Sp7-cre: 5′-TACCAGAAGCGACCACTTGAGC-3′; 5′-CGCCAAGAGAGCCTGGCAAG-3′; 5′-GCACACAGACAGGAGCATCTTC-3′ (wild type = 263 bp, heterozygote = 445 bp and 263 bp, homozygote = 445 bp).

### 2.17 Statistics

All results are presented as the mean ± SD. Comparisons between two groups were analyzed using a two-tailed, unpaired Student’s t-test. One-way Anova followed by Tukey’s post hoc test was used when the data involved multiple group comparisons. GraphPad PRISM v.10.0.0 was used for statistical analysis.

## 3 Results

### 3.1 Primary cilia are critical for growth plate development and chondroblast

#### 3.1.1 Differentiation

**Fig. 1:**
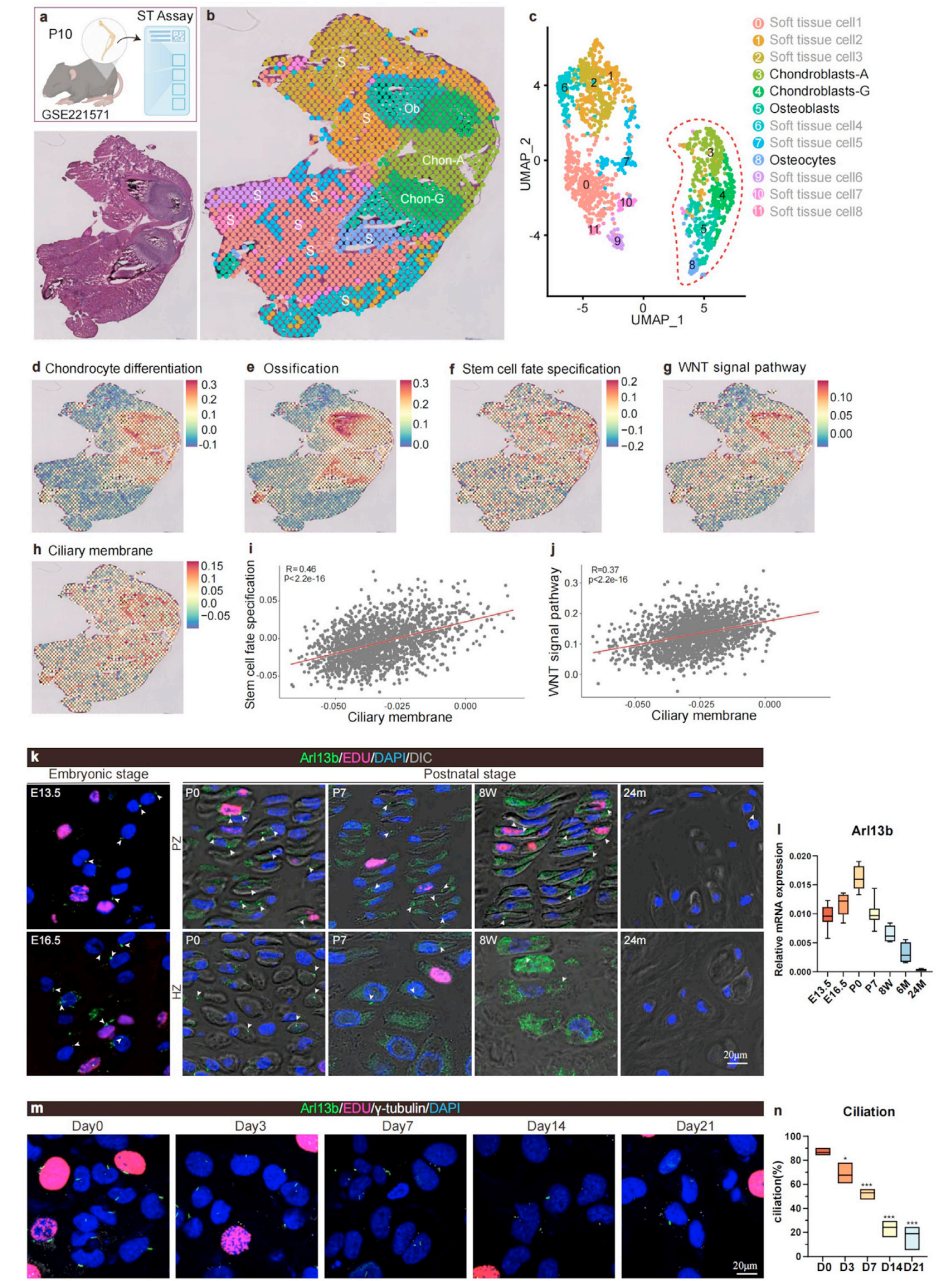
**Cilia are closely related to growth plate chondrocyte differentiation.** (a) Experimental scheme. (b & c) *Re*-analysis and clustering of the Stereo-seq data. (d-h) Spatial visualization of different module scores in the P10 femur sample. (i & j) correlation analysis of Ciliary membrane module and Stem cell fate specification module as well as Wnt signaling pathway module. (k) Immunofluorescent images of femur growth plate in WT mice from embryonic stage to aging stage. The arrows point to the primary cilia. (l) qPCR analysis of Arl13b expression in femur growth plate from embryonic stage to aging stage. (m) Immunofluorescent images of primary cilia and EDU in primary chondroblasts at different time point after chondrogenic differentiation induction (*n* = 6). (n) Ciliation of the primary chondroblasts at different differentiation time point which was calculated by Image J. PZ: proliferating zone; HZ: hypertrophic zone. *P < 0.05, ^⁎⁎⁎^*P* < 0.001.

Growth plates mediate the development of long bones [33,34]. To investigate the role of primary cilia, a specialized organelle, we reanalyzed spatial transcriptome data of P10 mouse femurs from an open-source database [31] (Fig. 1a). After quality control and the removal of doublets, we identified 12 cell subpopulations (Fig. 1b). Based on their spatial positions, we defined these subpopulations as chondroblasts, osteoblasts, osteocytes, and soft tissue cells of long bones (Fig. 1c). We mapped the gene ontology (GO) modules related to chondroblast differentiation, ossification, stem cell fate specification, and the Wnt signaling pathway (Fig. 1d-h). We found that the expression patterns of these GO terms, which are closely related to long bone development, were similar to those of ciliary membrane terms. Correlation analysis revealed that the ciliary membrane GO term module was highly correlated with stem cell fate specification and the Wnt signaling pathway (Fig. 1i and j).

To further investigate the role of primary cilia at various stages of long bone development, we analyzed the growth plates of mice from embryonic to elderly stages (24 months) using immunofluorescence staining (Fig. 1k). The results showed that the specific key gene Arl13b in the ciliary membrane was widely expressed in all layers of the growth plates from embryonic to young adult stages but was rarely observed in the aged. qPCR results were consistent with the staining results (Fig. 1l). Additionally, we conducted *in vitro* chondrogenic induction on primary chondrocytes from growth plates and detected the expression of Arl13b at different stages of induction. Immunofluorescence staining and qPCR results showed that the expression of the primary cilia marker gradually decreased as differentiation progressed (Figs. 1m, n and S1).

These findings indicate that primary cilia may mediate bone development by regulating cells within the long bone growth plate.

### 3.2 Inhibition of primary cilia formation in growth plate chondroblasts disrupts long bone development and signaling network

**Fig. 2:**
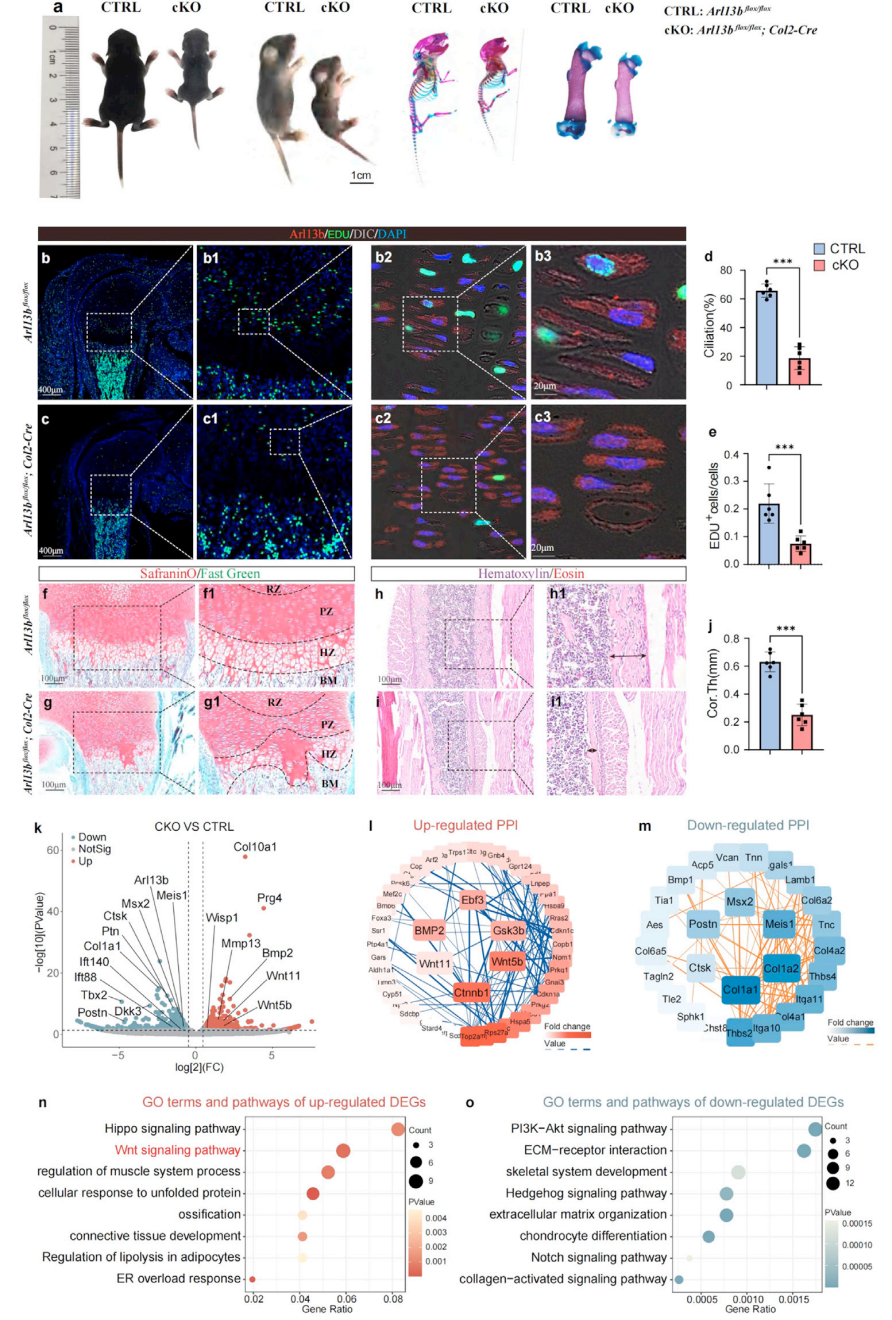
**The inhibition of primary cilia signaling in growth plate chondrocytes impairs chondrocytes proliferation and disrupts long bone development.** (a) Gross appearance and Alcian blue/ Alizarin red staining of Arl13b *^flox/flox^* and Arl13b *^flox/flox^*; Col2-Cre mice at 2 weeks of age. (b & c) Immunofluorescent images of femur growth plate of Arl13b *^flox/flox^* and Arl13b *^flox/flox^*; Col2-Cre mice at 2 weeks of age. (d & e) The histogram showing the ciliation and frequency of EDU^+^ cells of two groups, which was calculated by Image J (*n* = 6) ^⁎⁎⁎^*P* < 0.001. (f-i) SafraninO/Fast Green and HE staining of Arl13b *^flox/flox^* and Arl13b *^flox/flox^*; Col2-Cre mice at 2 weeks of age. (j) The histogram showing the cortical bone thickness of two groups, which was calculated by Image J (*n* = 6) ^⁎⁎⁎^*P* < 0.001. (k) Volcano plot showing differentially expressed genes of CKO mice growth plate compared with the control mice. (l & m) The PPI network of up-regulated and down-regulated genes of CKO mice compared with the control mice. (n & o) The dot plot showing the GO terms and pathways of up and down regulated DEGs of CKO mice growth plate compared with the control mice. RZ: resting zone; PZ: proliferating zone; HZ: hypertrophic zone; BM: bone marrow.

To further investigate the role of primary cilia in long bone development, we constructed conditional knockout (CKO) mice by crossing Arl13b*^flox/flox^* mice with Col2-cre mice, leading to the specific knockout of primary cilia in chondrocytes. More detailed targeting strategy and the primary cilia occurrence in both control and CKO mice were shown in Fig. S2. Two weeks after birth, gross observation and Alizarin Red and Alcian Blue staining revealed that the body length of CKO mice was significantly shorter than that of control mice, and the degree of long bone mineralization was lower (Fig. 2a). Immunofluorescence staining showed that the proportion of EDU^+^ cells and the frequency of primary cilia in the growth plates of CKO mice were lower (Fig. 2b-e). Safranin O/Fast Green staining indicated abnormal growth plate structure in CKO mice, characterized by disordered proliferative and hypertrophic layers (Fig. 2f and g). Hematoxylin/Eosin (HE) staining revealed a significant thinner cortical bone thickness in CKO mice (Fig. 2h-j).

Transcriptome sequencing analysis of growth plates from control and CKO mice demonstrated significant upregulation of genes related to the Wnt signaling pathway, such as Wnt11 and Wnt5b, and genes associated with terminal differentiation of hypertrophic chondrocytes, such as Col10a1 and Mmp13, in CKO mice compared to controls. Conversely, CKO mice showed significant downregulation of stem cell markers, including Meis1, Msx1, Postn, and primary ciliary key genes such as Arl13b and IFT140 (Fig. 2k). Protein-protein interaction (PPI) network analysis of upregulated and downregulated genes revealed that CKO mice upregulated key proteins in the Wnt signaling pathway (Fig. 2l) while downregulating key proteins in cellular stemness and chondrogenesis (Fig. 2m). GO and Kyoto Encyclopedia of Genes and Genomes (KEGG) enrichment analysis of downregulated genes in CKO mice indicated significant upregulation of the Wnt and Hippo signaling pathways, as well as pathways related to connective tissue development (Fig. 2n). Conversely, GO terms and signaling pathways related to skeletal system development, such as chondrogenesis and ECM−receptor interaction, were downregulated (Fig. 2o).

Together, these results confirm the critical importance of primary cilia in long bone development. Inhibition of primary cilia formation disrupts long bone development, likely due to the disruption of the signaling network within growth plate cells.

### 3.3 Inhibition of primary cilia formation in growth plate SSCs disrupts cell proliferation, differentiation and activity *in vitro*

**Fig. 3:**
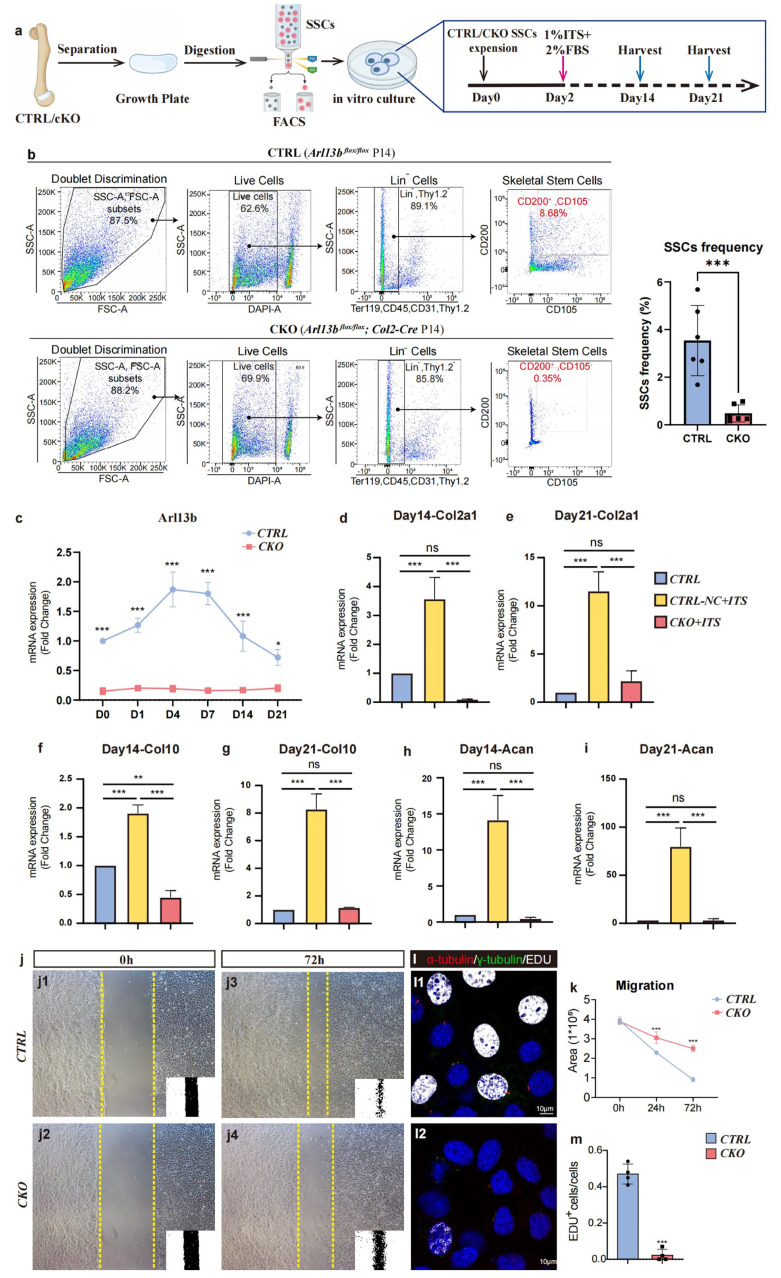
**Primary cilia in growth plate SSCs affects cell proliferation, differentiation and activity *in vitro*.** (a) Experimental scheme. (b) The FCM results showing the frequency of skeletal stem cells (SSCs) in growth plates of the control and the CKO mice at P14 (*n* = 6) ^⁎⁎⁎^*P* < 0.001. (c) The line chart showing the Arl13b expression of two groups during the chondrogenetic induction (*n* = 6) *P < 0.05, *^⁎⁎⁎^P* < 0.001. (d-i) The histogram showing the expression of Col2a1, Col10 and Acan among three groups at day14 and day21 after induction (*n* = 3) ns, no statistical significance, ^⁎⁎^*P* < 0.01, ^⁎⁎⁎^*P* < 0.001. (j) A scratch-wound assay was performed on growth plate primary skeletal stem cells grown to confluency of the two groups. Images were taken at the start of the experiment (0 h) and 72 hours later (72 h). (k) Quantification of migration by measuring the width of the cell-free zone at the time of the scratch (0 hours) and 72 hours after the scratch (*n* = 6) ^⁎⁎⁎^*P* < 0.001. (l) Immunofluorescent images of growth plate primary skeletal stem cells of the two groups. (m) The histogram showing the frequency of EDU^+^ cells of the two groups, which was calculated by Image J (*n* = 4) ^⁎⁎⁎^*P* < 0.001.

Furthermore, we sought to investigate the role of primary cilia in determining cell fate *in vitro*. We separated WT and CKO mice growth plates at P14 and obtained primary SSCs through flow cytometry (FCM) [35]. Following *in vitro* expansion, chondrogenic differentiation was induced, and chondrogenic-related markers were assessed at 14 and 21 days (Fig. 3a). The FCM results showed that the frequency of skeletal stem cells in the CKO mice growth plate was significantly lower than in the control (Fig. 3b). qPCR results indicated that primary skeletal stem cells from CKO mice expressed significantly lower levels of Arl13b compared to control mice throughout chondrogenic differentiation (Fig. 3c). Additionally, the expressions of cartilage-related genes Col2a1, Col10, and Acan were significantly suppressed in the middle and late stages of differentiation (Fig. 3d-i). All results above were consistent with the transcriptome sequencing results (Fig. 2d-i). Moreover, migration experiments confirmed that knocking out Arl13b can significantly inhibit cell migration (Fig. 3j and k). Immunofluorescence staining experiments showed that knocking out Arl13b can inhibit cell proliferation, which is consistent with *in vivo* immunofluorescence staining results (Fig. 3l and m). The above results propose that the key gene of primary cilia can inhibit the important biological processes of proliferation, differentiation and migration of skeletal stem cell/progenitor within growth plates.

### 3.4 Inhibition of primary cilia formation disrupts normal mitosis and asymmetric division in growth plate SSCs

**Fig. 4:**
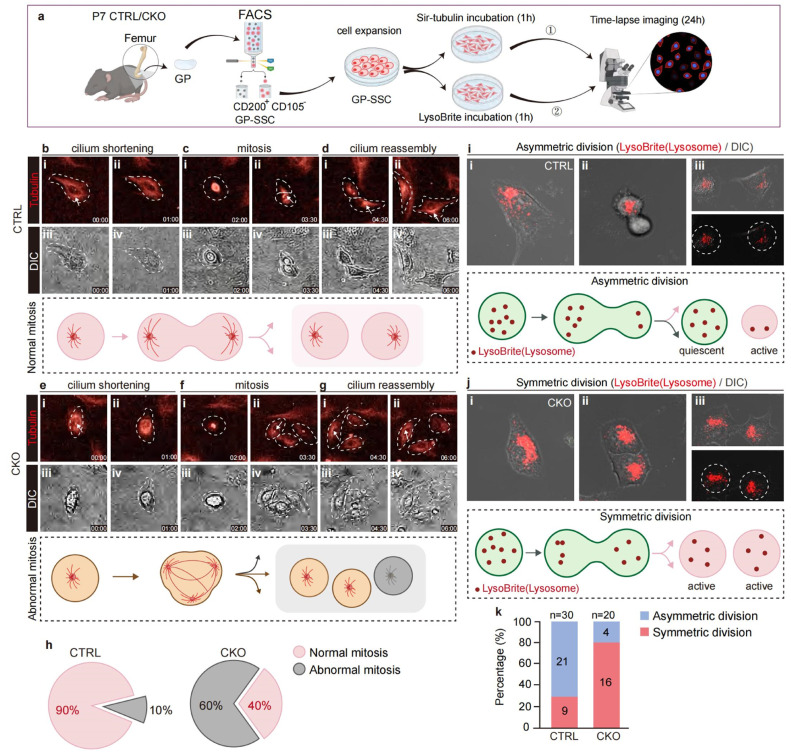
**Disruption of the cilia of skeletal stem cells in the growth plate of long bones can lead to decreased stemness and mitotic disorders.** (a) Experimental scheme. (b-d) Time-lapse imaging showing the Sir-tubulin probe distribution in control mice cells during cell division. i, ii. Immunohistochemical staining imaging, iii, iv. DIC imaging. (e-f) Time-lapse imaging showing the Sir-tubulin probe distribution in CKO mice cells during cell division. i, ii. Immunohistochemical staining imaging, iii, iv. DIC imaging. (h) Pie chart showing the percentage of different types of cell division in two groups of mice. (i & j) Time-lapse imaging showing the LysoBrite probe distribution in two groups of cells during cell division. i- iii. Different cell division stages during mitosis. (k) The histogram showing the percentage of different types of cell division in two groups of mice.

The SSCs within the growth plate are essential for maintaining structural integrity and regulating further bone development. Cell division involves two types: asymmetric and symmetric division. The fate decisions of SSC daughter cells can, therefore, be viewed as binary, resulting in three potential combinations: both daughters adopting the same fate and differentiating, or only one daughter differentiating while the other retains a stem cell identity. The resting zone (RZ) houses a population of SSCs that utilize asymmetric division to maintain the structure of the RZ, while also differentiating into chondroblasts to contribute to the proliferating zone (PZ). In this study, we aimed to determine whether primary cilia play a critical role in maintaining SSC stemness by regulating cell fate. Time-lapse imaging was employed to observe cell division in both control and CKO SSCs (Fig. 4a). Firstly, the Sir-tubulin was used to investigate if knockout of the primary cilia would affect the normal cell mitosis. The results indicated that most control SSCs underwent normal mitosis, evidenced by one cell dividing into two daughter cells. During normal mitosis, primary cilia exhibited cycles of shortening and reassembly (Fig. 4b-d). In contrast, the assembly of primary cilia was markedly impaired in CKO SSCs (Fig. 4e-f). Additionally, abnormal cell divisions were frequently observed among CKO SSCs, accounting for 60% of all events (Fig. 4h). We subsequently employed the lysosome reporter probe LysoBrite to assess lysosomal distribution in SSCs, which serves as an indicator of asymmetric versus symmetric division [36]. The results demonstrated that SSCs of control mice were more likely to undergo asymmetric division, while the majority of SSCs from CKO mice exhibited symmetric division (Fig. 4i-k), suggesting that control SSCs were more effective at maintaining stemness. These findings propose a critical role for primary cilia in regulating SSC stemness within the growth plate.

### 3.5 Inhibition of primary cilia formation exhausts stem cells and accelerates mineralization in the growth plate

**Fig. 5:**
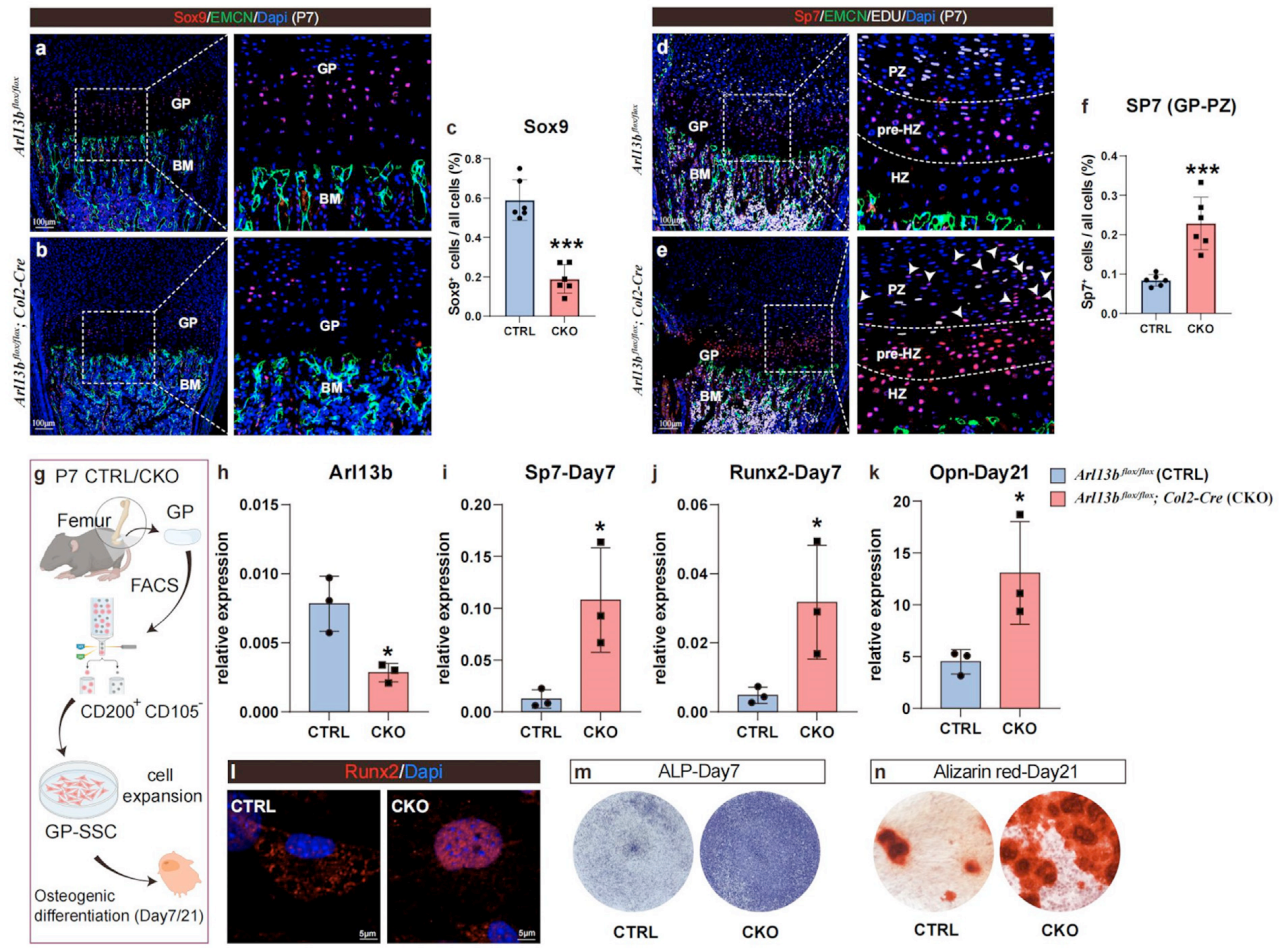
**Blocking primary cilia signaling reduces Hedgehog (Hh) and leads to excessive mineralization within the growth plate.** (a & b) Immunofluorescence staining showing the Sox9^+^ cells distributed in the growth plate structures of the two group at P7. (c) The histogram showing the expression of Sox9 in two groups at P7 (*n* = 6). ^⁎⁎⁎^*P* < 0.001. (d & e) Immunofluorescence staining showing the Sp7^+^ cells distributed in the growth plate structures of the two group at P7. (f) The histogram showing the expression of Sp7 among PZ in two groups at P7 (*n* = 6). ^⁎⁎⁎^*P* < 0.001. (g) Experimental scheme. (h-k) The histogram showing the expression of Arl13b, Sp7, Runx2 and Opn in two groups at day7 and day21 after induction (*n* = 3), *P < 0.05. (l) Immunofluorescence staining showing the Runx2 signal distribution in cell nuclear in CKO SSCs, while there was more cytoplasmic localization in control SSCs. (m & n) The ALP and Alizarin red staining in two groups at day7 and day21 after induction.

Previous studies have demonstrated that chondroblasts require a Wnt inhibitory niche to balance cell stemness and differentiation during bone development [37]. Building on this, we explored whether the knockout of primary cilia in SSCs compromises stemness by inducing excessive differentiation. Immunofluorescence staining revealed a significant decrease in the percentage of Sox9^+^ cells in the growth plate RZ of CKO mice compared to controls (Fig. 5a-c). Meanwhile, the Sp7^+^ cells appeared more frequently in the pre-hypertrophic zone (HZ) of CKO mice, with some even present in the PZ, indicating premature differentiation of chondroblasts in CKO mice (Fig. 5d-f). We isolated GP-SSCs from both control and CKO mice at P7 using FCM and applied osteogenic differentiation induction *in vitro* (Fig. 5g). The qPCR results confirmed the significant reduction in Arl13b expression in CKO SSCs (Fig. 5h). During osteogenic differentiation, the osteogenic markers, such As *sp7*, Runx2 and Opn, were significantly induced in CKO mice (Fig. 5i-k). Immunofluorescence staining further demonstrated the nuclear localization of Runx2 in CKO SSCs, while control SSCs exhibited more cytoplasmic localization (Fig. 5l). These results also indicated overactivation of Runx2 signaling in CKO SSCs. Additionally, ALP and Alizarin red staining on Day 7 and 21 indicated excessive mineralization in CKO mice (Fig. 5m and n).

Primary cilia are critical for the Indian Hedgehog (IHH) signal pathway, as most receptors and ligands are localized to the primary cilia membrane. IHH signaling is also vital for differentiating chondroblasts in the PZ into hypertrophic chondrocytes within the HZ. Spatial transcriptome analysis also indicated that the genes related to HH signaling were widely distributed in mouse long bone at P10 (Fig. S3a-d). Thus, we aimed to determine whether excessive mineralization in CKO mice was mediated by the overactivation of IHH signaling. However, the GO analysis from RNA sequencing indicated the downregulation of IHH signaling (Fig. 2o). Immunofluorescence staining also revealed a reduced number of IHH^+^ cells in the growth plate of CKO mice (Fig. S4a-4c). The immunofluorescence staining indicated that the Smo signal, which suggested the IHH signaling, was not absent in the primary cilia of CKO SSCs (Fig. S4d, 4e). The primary cilia in CKO cells exhibited shorter lengths, suggesting a phenomenon akin to ``cilia excision'' (Fig. S4f, 4 *g*), which may partially explain the attenuated IHH signaling in CKO cells. This leads us to postulate that the remaining primary cilia in CKO mice retain the capacity to mediate Hh signaling through preserved SMO membrane localization, although the efficiency of signal transmission may have been compromised. These results indicate that the excessive mineralization within the CKO growth plate may be mainly attributed to the overactivation of Wnt signaling mediated by primary cilia. However, further experiments need to be carried out to further explore the difference between Hh and Wnt signaling within our CKO mice.

### 3.6 Inhibition of primary cilia formation leads to aberrant Wnt signaling activation and ECM degradation in the growth plate

**Fig. 6:**
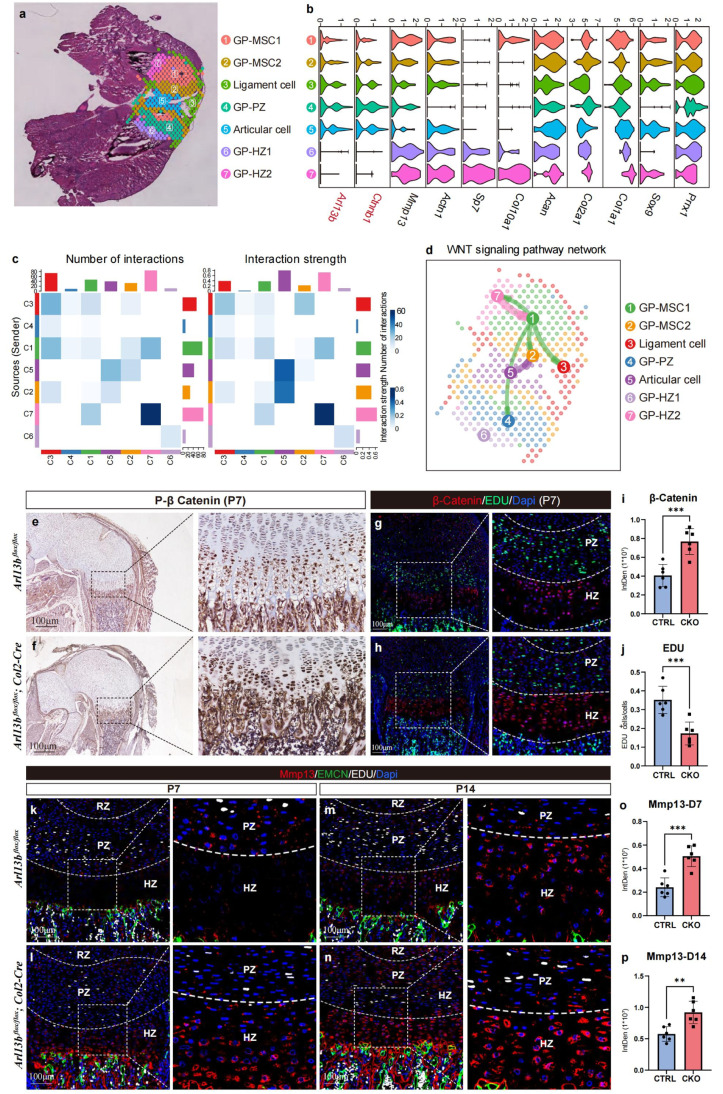
**Blocking cilia formation leads to abnormal activation of Wnt in chondrocytes of cartilage growth plate.** (a) *Re*-analysis and clustering of the Stereo-seq data within growth plate and arthrosis. (b) Violin plots showing the expression levels of representative marker genes across the 7 clusters. (c) Heatmap showing the signal pathway interaction across 7 subsets, which was calculated by cell chat. (d) WNT signaling pathway network across 7 subsets calculated by cell chat. (e & f) Immunohistochemical staining showing the expression of p-β-Catenin of both control and CKO mice femur at P7. (g & h) Immunofluorescent staining showing the expression of β-Catenin of both control and CKO mice femur at P7. (i & j) The histogram showing the expression of β-Catenin and the frequency of EDU^+^ cells of the two groups (*n* = 6) ^⁎⁎⁎^*P* < 0.001. (k-n) Immunofluorescent staining showing the expression of Mmp13 of both control and CKO mice femur at P7 and P14 respectively. (o & p) The histogram showing the expression of Mmp13 of the two groups (*n* = 6) ^⁎⁎^*P* < 0.01, ^⁎⁎⁎^*P* < 0.001.

To delve deeper into the specific mechanism by which primary cilia regulate growth plates, we conducted further analysis on spatial transcriptome data from P10 mice. After excluding doublets from the growth plate, articular chondrocytes, and surface ligament cells, we identified 7 distinct cell subpopulations (Fig. 6a). Due to the additional spatial information as well as the relatively low sequencing saturation of the spatial transcriptome data, the clustering results of the femur and tibia did not perfectly match. We adjusted the resolution so that the bone marrow and growth plate-related clusters were preserved at both sites. Examination of marker genes within each subpopulation revealed similarities in the expression patterns of the primary ciliary key gene Arl13b and the Wnt signaling key gene Ctnnb1, particularly enriched in the growth plate MSCs (which contain SSCs of GP-RZ) and PZ (Fig. 6b). Subsequent analysis of signal interactions among various cell subsets using Cell Chat unveiled extensive signaling crosstalk within growth plate subsets (Fig. 6c), with the Wnt signaling pathway forming a closely interconnected network, centered around growth plate MSCs (Fig. 6d).

To further corroborate the role of primary cilia in mediating the Wnt signaling pathway in growth plates, we assessed the expression of the key molecule β-catenin using immunohistochemistry (Fig. 6e and f) and immunofluorescence staining (Fig. 6g and h) in control and CKO mice growth plates. Our findings indicated a significant upregulation of β-catenin expression as well as the EDU^+^ cells' frequency in the proliferative and hypertrophic layers of growth plates in CKO mice (Fig. 6i and j). Additionally, in line with sequencing data suggesting upregulation of matrix metalloproteinase expression in CKO mice, we examined the expression of Mmp13 in the growth plates of P7 and P14 mice using immunofluorescence, revealing a significant upregulation in CKO mice (Fig. 6k-p).

These results collectively confirm that primary cilia mediate the interaction of Wnt signaling pathways in the long bone growth plate. Disruption of primary cilia function leads to aberrant activation of the Wnt signaling pathway and subsequent excessive secretion of Mmp13, likely contributing to the structural abnormalities observed in knockout mice. On the other hand, Wnt signaling mediates the asymmetric cell division (ACD) during embryonic development [38], which also partially explains the abnormal cell division and decreased ACD in CKO SSCs during time-lapse imaging.

### 3.7 Targeted inhibition of the Wnt signaling pathway partly rescues abnormal long bone development in CKO mice

**Fig. 7:**
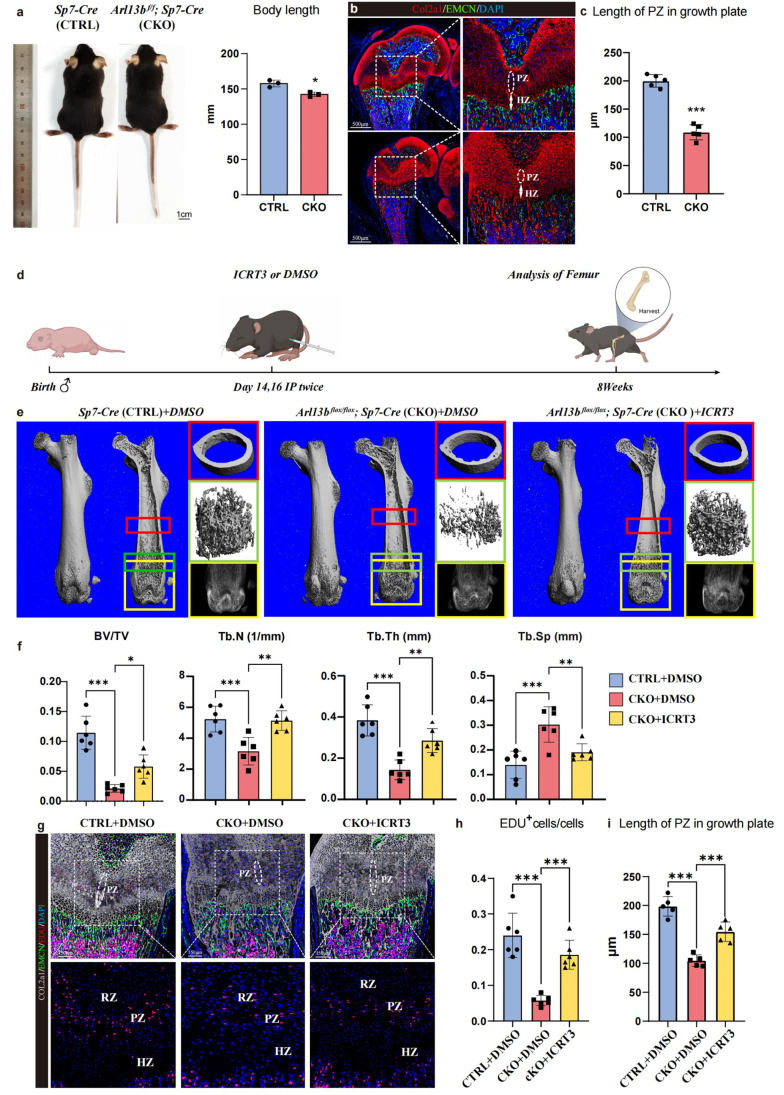
**Targeted inhibition of the Wnt signaling pathway partly rescued abnormal bone formation.** (a) Gross appearance of Sp7-Cre and Arl13b *^flox/flox^*; Sp7-Cre mice at 8 weeks of age (*n* = 3) ns, no statistical significance, *P < 0.05. (b) Immunofluorescence staining showing the growth plate structure of the two groups. (c) The histogram showing the length of PZ in growth plate between control and cKO mice (*n* = 5) ^⁎⁎⁎^*P* < 0.001. (d) Experimental scheme. (e) µ-CT imaging of bone in coronal section, cross section, reconstruction of trabecular bone and the X-ray of the metaphysis among the three groups. (f) The histogram showing the bone volume/ tissue volume, trabecular bone number, trabecular bone thickness and trabecular bone separation (*n* = 6) *P < 0.05, ^⁎⁎^*P* < 0.01, ^⁎⁎⁎^*P* < 0.001. (g) Immunofluorescent staining showing the structure and the EDU^+^ cells in the growth plate among the three groups. (h) The histogram showing the frequency of EDU^+^ cells among the three groups. (i) The histogram showing the length of PZ in growth plate among the three groups (*n* = 5 or 6) ^⁎⁎⁎^*P* < 0.001.

However, complete knockout of primary cilia throughout the growth plate layer in Arl13b *^f/f^* Col2-cre mice results in mortality at 2–3 weeks, potentially due to impaired thoracic development, rendering this model unsuitable for subsequent rescue experiments. The detailed phenotypic comparisons between the two groups are presented in Fig. S5. To address this limitation, we selectively disrupted cilia in hypertrophic cartilage and preosteoblasts by crossing Arl13b*^flox/flox^* with Sp7-cre mice, which showed milder phenotype during development. The specific distribution of Sp7^+^ cells within the growth plate was confirmed through analysis of immunofluorescence staining and *Sp7-cre; tdTomato* mice (Fig. S6), which was consistent with the prior research [39]. The phenotype of control and CKO mice among the lone bone development were shown in Fig. S5. Remarkably, the CKO mice survived to adulthood, albeit with shorter body length compared to control mice (Fig. 7a). Immunofluorescence staining revealed significantly decreased thickness of each growth plate layer in CKO mice at 8 weeks (Fig. 7b and c). HE staining also showed reduced cortical thickness (Fig. S7a).

We administered the DMSO and Wnt inhibitor ICRT3 into the joint cavity of 2-week-old control and CKO mice to investigate the potential therapeutic efficacy of Wnt signaling inhibition. The injection was applied twice on postnatal days 14 and 16 of the mice. Subsequently, we analyzed femoral cortical bone and trabecular phenotype using µ-CT after 6 weeks (Fig. 7d). Our findings demonstrated that CKO mice exhibited lower bone mass, decreased trabecular bone number, and reduced trabecular thickness compared to control mice. However, treatment with ICRT3 partially rescued these phenotypic abnormalities in CKO mice (Fig. 7e and f). HE staining further validated that the ICRT3 could improve cortical bone thickness in CKO mice (Fig. S7b). Moreover, Immunofluorescence staining revealed a lower proportion of EDU^+^ cells in the growth plate of CKO mice compared to controls, while ICRT3 treatment increased the proportion of EDU^+^ cells in the growth plate of CKO mice as well as partially rescued the growth plate decreased thickness in CKO mice (Fig. 7 g-i).

These results underscore the pivotal role of the Wnt signaling pathway mediated by primary cilia across all layers of growth plate cells. Moreover, targeting this signaling pathway holds promise for partially rescuing the phenotype observed in CKO mice, highlighting its therapeutic potential in mitigating abnormal bone development associated with primary cilia dysfunction.

## 4 Discussion

Primary cilia are integral to the regulation of cellular signaling in a variety of tissues, with their presence in cartilage being notably higher compared to other regions of the long bone [40,41]. Previous studies have demonstrated that primary cilia are present in all layers of cells within the growth plate [42,43], and their orientation is highly synchronized with key physiological processes such as cell proliferation, differentiation, and survival [44,45]. However, the precise role of primary cilia in bone development and their involvement in the regulatory signaling network remain poorly understood. Our study provides compelling evidence linking primary cilia to stem cell fate regulation and Wnt signaling within the growth plate, as shown through spatial transcriptomics. Immunofluorescence and qPCR analyses of growth plates across developmental stages—ranging from embryonic to senescent mice—revealed that primary cilia are consistently present in chondroblasts throughout development. Conditional knockout (CKO) of the key ciliary gene Arl13b in the growth plate resulted in significant structural anomalies that impaired long bone development in CKO mice. These findings underscore the essential role of primary cilia in maintaining growth plate homeostasis.

To investigate the molecular mechanisms through which primary cilia influence long bone development, we performed transcriptomic profiling of control and CKO mice. Our analysis identified a significant upregulation of the Wnt signaling pathway in CKO mice. Spatial transcriptomics further revealed the interaction of Wnt signaling across different layers of the growth plate. These results suggested that Wnt signaling plays a central role in coordinating cellular activities within the growth plate, such as cell proliferation and differentiation. Previous studies have shown that Wnt/β-catenin signaling regulates the differentiation of progenitor cells into osteoblasts or chondrocytes in the developing skeleton [46]. Disruption of β-catenin in mouse embryos leads to ectopic chondrocyte formation at the expense of osteoblast differentiation, whereas ectopic activation of Wnt signaling suppresses chondrocyte differentiation and enhances ossification [47]. These findings are consistent with our own data, both at the level of immunofluorescence and RNA sequencing. To further validate the role of Wnt signaling in bone development, we administered local injections of Wnt signaling inhibitors into the growth plates of CKO mice, which partially rescued the impaired bone development phenotype. These results confirm that primary cilia, through their regulation of Wnt signaling, are critical for maintaining the homeostasis of the growth plate and ensuring normal long bone development.

However, several limitations must be acknowledged. As indicated by prior research, Sp7-cre has been demonstrated to label chondroblasts within the prehypertrophic, hypertrophic, and partial proliferative zones of the growth plate [39,48], which appears consistent with our immunofluorescence staining and Sp7 lineage tracing results. On the other hand, *Arl13b^f/f^-Col2* CKO mice revealed severe thoracic skeletal dysplasia culminating in perinatal lethality (2-week-old), which made them not a suitable rescue experimental model. Consequently, the rescue experiments were carried out on *Arl13b^f/f^; Sp7-cre* CKO mice instead of *Arl13b ^f/f^; Col2-cre* CKO mice, despite the similar phenotypic manifestations in growth plate structure and cortical bones. However, we recognize that the phenotypic manifestations in *Arl13b^f/f^; Sp7-cre* CKO mice are likely also associated with osteoblast differentiation processes, which was inconsistent with that in *Arl13b ^f/f^; Col2-cre* mice. Thus, future studies employing *Col2-creERT2* or *Col10a1-cre* CKO mouse models would be rational in specifically clarifying the functional contributions of primary cilia in the regulation of growth plate cells. In particular, it would be better to apply *Prrx1-creERT2* or *Pthrp-creERT2* CKO mice to specifically focus on the research on the growth plate SSCs, as SSC located at the upstream of the chondrogenesis differentiation trajectory within the growth plate. Additionally, while our study suggests a critical role for primary cilia in modulating stem cell fate, the exact mechanisms by which primary cilia regulate stem cell activity and asymmetric cell division through Wnt signaling remain unclear. Further investigations are needed to elucidate how primary cilia interact with other key signaling pathways, such as Hedgehog signaling, to regulate long bone development.

In summary, our findings highlight the pivotal role of primary cilia in maintaining the growth plate homeostasis, dynamically orchestrating the spatiotemporal activation of Wnt signaling to preserve the microenvironmental equilibrium. Their dysfunction triggers network-wide signaling imbalance (e.g., Wnt hyperactivation) and aberrant stem cell mitotic activity. Meanwhile, we employed different developmental stage mouse models to demonstrate the critical roles of primary cilia throughout long bone development, including the cartilage patterning during embryogenesis (E13.5-P0), proliferation-hypertrophic transitions postnatally (P7–2 weeks), and adult bone homeostasis maintenance (8 weeks to 24 months) [1,49,50]. These results deepen our understanding of the complex signaling network that regulates long bone development and may provide valuable insights for the development of therapeutic strategies targeting bone developmental disorders.

## 5 Conclusion

In conclusion, our study identifies primary cilia as critical regulators of the growth plate signaling network, essential for maintaining the proper development of long bones. Disruption of primary cilia results in aberrant Wnt signaling activation and abnormal mitotic activity in SSCs, leading to impaired growth plate structure and defective bone elongation. These findings underscore the essential role of primary cilia in skeletal development and highlight their potential as therapeutic targets in clinical bone disorders. Further research into the mechanistic pathways mediated by primary cilia will provide valuable insights into their role in skeletal biology and inform future therapeutic strategies for skeletal developmental diseases.

## Ethics approval and consent to participate

The animal studies protocols were approved by the Animal Welfare Committee of Tongji University with Protocol numbers: [2022]-DW-06.

## Declaration of generative AI and AI-assisted technologies in the writing process

During the preparation of this work the authors used ChatGTP in order to improve language and readability. After using this tool, the authors reviewed and edited the content as needed and takes full responsibility for the content of the publication.

## Data availability

Data are available from the corresponding authors upon reasonable request.

## CRediT authorship contribution statement

**Lei Zhang:** Conceptualization, Data curation, Formal analysis, Software, Visualization, Writing – original draft. **Xiaoqiao Xu:** Data curation, Formal analysis, Visualization. **Dike Tao:** Data curation, Investigation, Project administration, Visualization. **Xinyu Li:** Formal analysis. **Pingping Niu:** Methodology, Validation. **Xuyan Gong:** Project administration, Supervision. **Gongchen Li:** Investigation, Resources. **Mengfei Yu:** Investigation. **Yao Sun:** Conceptualization, Funding acquisition, Resources, Supervision, Writing – original draft.

## Declaration of competing interest

The authors declare that they have no conflicts of interest in this work.

## Appendix Supplementary materials

Image, application 1

Image, application 2
