# Supplementary material for: Primary cilia in growth plates orchestrate long bone development
Source: Fundam Res. 2025 May 3;5(5):2368–81. doi: 10.1016/j.fmre.2025.04.014 (PMC12848162; doi:10.1016/j.fmre.2025.04.014)
Supplement: Supplementary file 1 [file mmc1.docx]

**Supplemental information**

**Supplementary Table 1.** Catalog numbers for the antibodies.

| REAGENT or RESOURCE | SOURCE | IDENTIFIER |
| --- | --- | --- |
| Anti-mouse CD45-APC (Clone: 30-F11) | Biolegend | Cat# 103112;  RRID AB_312977 |
| Anti-mouse Ter119-APC (Clone: TER-119) | Biolegend | Cat# 116212;  RRID AB_313713 |
| Anti-mouse CD31-APC (Clone: MEC13.3) | Biolegend | Cat# 102510;  RRID AB_312917 |
| Anti-mouse CD105-PE/Cy5 (Clone: MJ7/18) | BioLegend | Cat# 120428;  RRID AB_2819845 |
| Anti-mouse CD200-PE/Cy7 (Clone: OX-90) | BioLegend | Cat# 123817;  RRID AB_2876444 |
| Anti-mouse Thy1.2-APC (Clone: 5E10) | BioLegend | Cat# 328124;  RRID AB_2561693 |
| Rabbit polyclonal anti-Col2a1 | Boster | Cat# BA0533; |
| Mouse monoclonal anti-γ-tubulin (Clone: GTU-88) | Sigma-Aldrich | Cat# T5326; |
| Mouse monoclonal anti-ace α-tubulin (Clone: 6-11B-1) | Sigma-Aldrich | Cat# T6793; |
| Mouse monoclonal anti-Smo | Proteintech | Cat# 66851-2-Ig |
| Rabbit monoclonal anti-Sox9 | Abcam | Cat# ab185966 |
| Rabbit monoclonal anti-Sp7 | Abcam | Cat# ab209484 |
| Rabbit monoclonal anti-Runx2 | Abcam | Cat# ab192256 |
| Rabbit polyclonal anti-Arl13b | Proteintech | Cat# 17711-1-AP |
| Rabbit polyclonal anti-Mmp13 | Proteintech | Cat# 18165-1-AP |
| Rabbit polyclonal anti-β-catenin | Proteintech | Cat# 51067-2-AP |
| Rabbit polyclonal anti-IHH | Abcam | Cat# ab39634 |
| Rat monoclonal anti-Endomucin (Clone: V.7C7) | Santa Cruz | Cat# sc-65495; |
| Donkey polyclonal anti-rabbit Alexa Fluor 488 | Invitrogen | Cat# A21206;  RRID AB_2535792 |
| Donkey polyclonal anti-rabbit Alexa Fluor 594 | Invitrogen | Cat# A32754;  RRID: AB_2762827 |
| Donkey polyclonal anti-rabbit Alexa Fluor 647 | Invitrogen | Cat# A31573;  RRID: AB_2536183 |
| Donkey polyclonal anti-mouse Alexa Fluor 488 | Invitrogen | Cat# A32766;  RRID: AB_2762823 |
| Donkey polyclonal anti-mouse Alexa Fluor 594 | Invitrogen | Cat# A32744;  RRID: AB_2762826 |
| Donkey polyclonal anti-Rat Alexa Fluor 488 | Invitrogen | Cat# A-48269;  RRID AB_2893137 |

| Gene name | Forward primer (5’-3’) | Reverse primer (5’-3’) |
| --- | --- | --- |
| *Gapdh* | TGTGTCCGTCGTGGATCTGA | CCTGCTTCACCACCTTCTTGA |
| *Arl13b* | CACCGGAAAAAGAATTCGGGGAATT | AAACAATTCCCCGAATTCTTTTTCC |
| *Col2a1* | CGAGTGGAAGAGCGGAGACT | AACTTTCATGGCGTCCAAGGT |
| *Col10a1* | TAAGAACGGCACGCCTACGA | TGATTGCACTCCCTGAAGCC |
| *Acan* | GAAGAGCCTCGAATCACCTG | ATCCTGGGCACATTATGGAA |
| *Alp* | CACGCGATGCAACACCACTCAGG | GCATGTCCCCGGGCTCAAAGA |
| *Sp7* | CCCACTGGCTCCTCGGTTCTCTCC | GCTGAAAGGTCAGCGTATGGCTTC |
| *Runx2* | TTACCTACACCCCGCCAGTC | TGCTGGTCTGGAAGGGTCC |
| *Opn* | CTGACCTCACAGATGCCAAGC | TGGTCTGATAGCTCGTCACAAG |

**Supplementary Table 2.** Primer sequences for RT-qPCR.

**Supplementary Table 3.** Hedgehog pathway-associated gene ontology modules.

| GO TERM | Gene |
| --- | --- |
| GOMF_PATCHED_BINDING | Dhh,Ihh,Ptch1,Shh,Ccnb1,Smo,Ccnb1-ps,Bbs1 |
| REACTOME_HEDGEHOG_LIGAND_BIOGEN | Psmc3,Shh,Psmd4,Psmb4,Ihh,Psmd2,Scube2,Ubc,Psmb1,Psma2,Psmd3,Psmd11,Psmb6,Derl2,Ubb,Erlec1,Rps27a,Psmc5,Psmd12,Sel1l,Psma6,Psmc1,Psmd6,Psmc6,Gpc5,Psmb5,Psme1,Dhh,Psmb8,Syvn1,P4hb,Psmd13,Psmd1,Psmb7,Psmd5,Psmd14,Psma7,Vcp,Psmb2,Psmc2,Psmd9,Psmd8,Psmc4,Psma1,Psmd10,Psmb10,Psma4,Psmf1,Psma8,Hhat,Psmd7,Disp2,Os9,Psme4,Notum,Psma3,Uba52rt,Psma5,Psmb3,Psmb11,Psme2b,Psme3,Psme2,Uba52 |
| REACTOME_HEDGEHOG_ON_STATE | Smo,Psmc3,Shh,Cul3,Psmd4,Psmb4,Ihh,Psmd2,Ubc,Psmb1,Psma2,Psmd3,Psmd11,Psmb6,Smurf2,Kif3a,Arrb1,Ubb,Rps27a,Psmc5,Psmd12,Psma6,Psmc1,Numb,Gli3,Ptch1,Psmd6,Psmc6,Psmb5,Psme1,Rbx1,Dhh,Psmb8,Csnk1a1,Grk2,Sufu,Gli1,Psmd13,Psmd1,Cdc73,Psmb7,Spopl,Psmd5,Psmd14,Psma7,Itch,Psmb2,Psmc2,Evc,Psmd9,Psmd8,Psmc4,Psma1,Psmd10,Psmb10,Psma4,Ulk3,Psmf1,Psma8,Cdon,Smurf1,Psmd7,Gas8,Gpr161,Psme4,Dzip1,Gli2,Evc2,Kif7,Gas1,Spop,Psma3,Arrb2,Hhip,Uba52rt,Psma5,Psmb3,Psmb11,Psme2b,Psme3,Psme2,Uba52 |
| REACTOME_SIGNALING_BY_HEDGEHOG. | Tubb6,Tulp3,Smo,Psmc3,Shh,Cul3,Prkacb,Prkaca,Adcy9,Psmd4,Psmb4,Ihh,Psmd2,Scube2,Ubc,Fuz,Psmb1,Psma2,Tubb1,Psmd3,Psmd11,Ift52,Psmb6,Smurf2,Kif3a,Derl2,Arrb1,Ubb,Erlec1,Adcy1,Rps27a,Prkar1a,Adcy3,Psmc5,Psmd12,Sel1l,Psma6,Psmc1,Numb,Gli3,Ptch1,Adcy2,Psmd6,Psmc6,Gpc5,Psmb5,Psme1,Adcy4,Adcy8,Rbx1,Gsk3b,Adcy5,Adcy6,Dhh,Tuba1b,Ift140,Psmb8,Csnk1a1,Syvn1,Grk2,P4hb,Sufu,Gli1,Psmd13,Prkar1b,Tuba4a,Psmd1,Cdc73,Adcy10,Psmb7,Spopl,Psmd5,Psmd14,Psma7,Itch,Vcp,Psmb2,Psmc2,Evc,Psmd9,Cul1,Ift122,Psmd8,Psmc4,Psma1,Psmd10,Adcy7,Psmb10,Psma4,Ulk3,Psmf1,Ift57,Rpgrip1l,Mks1,Ttc21b,Skp1,Psma8,Tubb4b,Hhat,Wdr19,Cdon,Ift172,Smurf1,Psmd7,Disp2,Gas8,Os9,Ofd1,Gpr161,Psme4,Dzip1,Notum,Tuba1c,Tubb2b,Dync2h1,Gli2,Evc2,Kif7,Gas1,Spop,Tubb2a,Psma3,Arrb2,Intu,Tubb3,Tubb4a,Hhip,Wdr35,Tuba3b,Tuba3a,Uba52rt,Psma5,Psmb3,Tuba1a,Psmb11,Psme2b,Psme3,Psme2,Uba52 |
