# Supplementary material for: Primary cilia in growth plates orchestrate long bone development
Source: Fundam Res. 2025 May 3;5(5):2368–81. doi: 10.1016/j.fmre.2025.04.014 (PMC12848162; doi:10.1016/j.fmre.2025.04.014)
Supplement: Supplementary file 2 [file mmc2.docx]

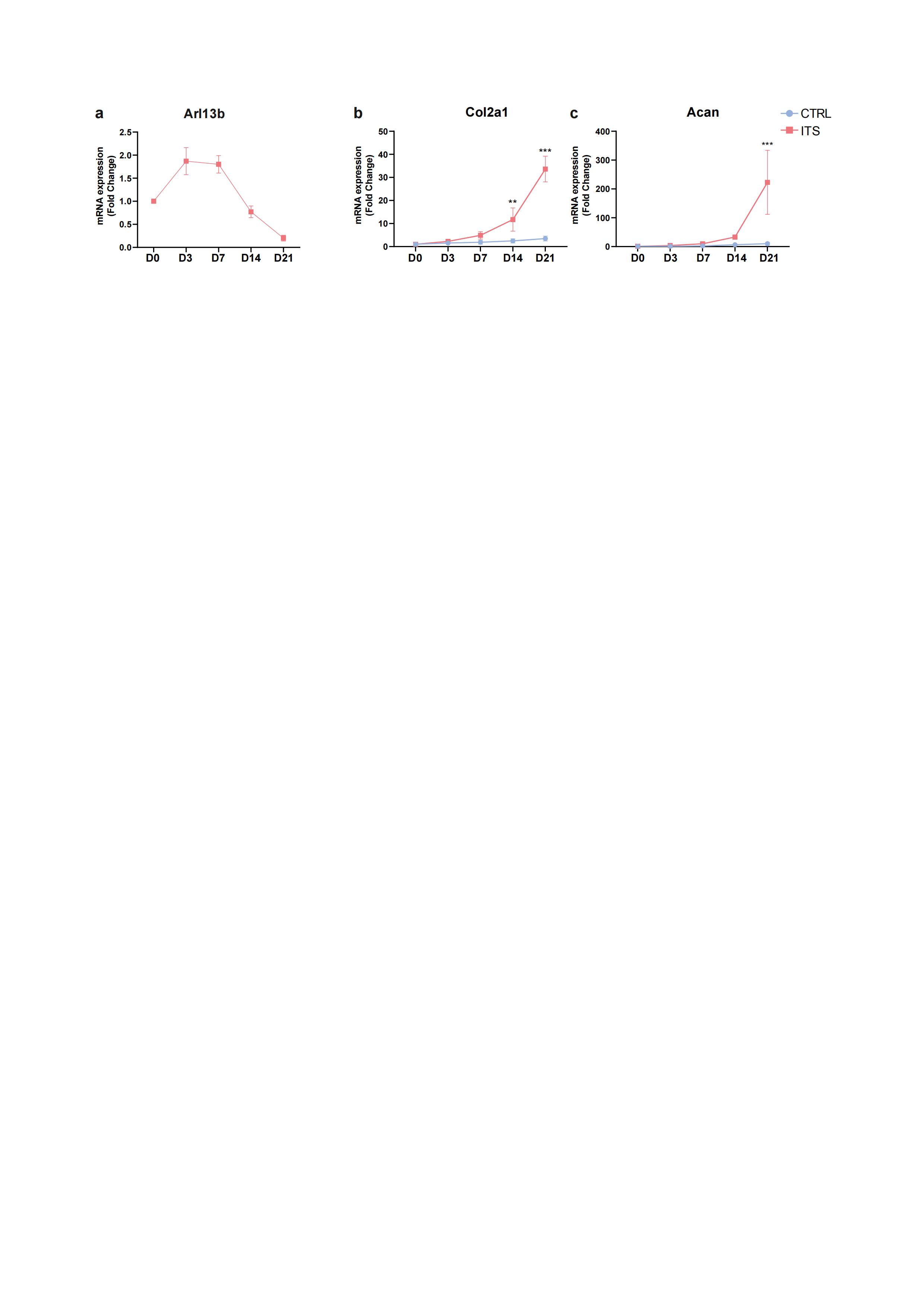
**Supplementary Figure legends**

**Supplementary Figure 1.** Expression of Arl13b and chondrogenic markers during *In Vitro* chondrogenesis induction. (a) The line chart showing the Arl13b expression of the primary chondroblasts during the chondrogenetic induction (n=6). (b, c) The line chart showing the expression of Col2a1 and Acan of both control (CTRL) and chondrogenetic induction (ITS) groups during the normal or chondrogenetic induction (n=3) **:P<0.01, ***:P < 0.001

**
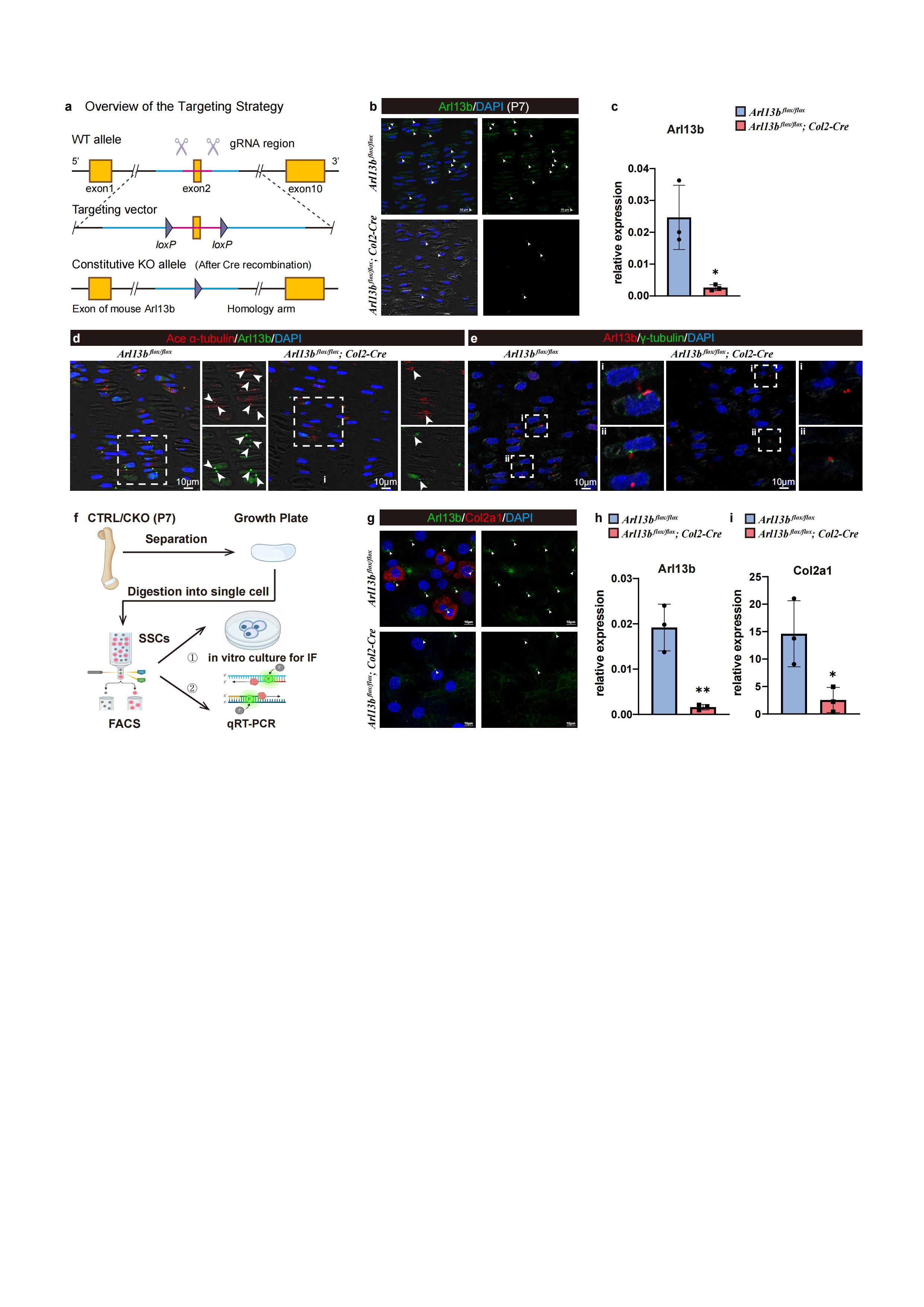
Supplementary Figure 2.** Primary cilia are critical for growth plate development and chondroblasts differentiation. (a) Targeting strategy of the Arl13b CKO mice. (b) Representative immunofluorescent images for ARL13B (green) expression in P7 control and Arl13b CKO mice. White arrows indicate primary cilia. Scale bar: 10 μm. (c) The histogram showing the ARL13B mRNA expression levels in primary growth plate SSCs of both control and Arl13b cKO mice (n=3). (d) Representative immunofluorescent images for Ace α-tubulin and Arl13b expression in P7 control and Arl13b CKO mice. White arrows indicate primary cilia. (e) Representative immunofluorescent images for Arl13b and γ-tubulin expression in P7 control and Arl13b CKO mice. (f) Experimental scheme. (g) Immunofluorescent images of growth plate primary skeletal stem cells cultured in vitro of the two groups. White arrows indicate primary cilia. (h & i) The histogram showing the ARL13B and Col2a1 mRNA expression levels of the two groups, which was calculated by Image J (n=3) *:P < 0.05, **:P < 0.01.


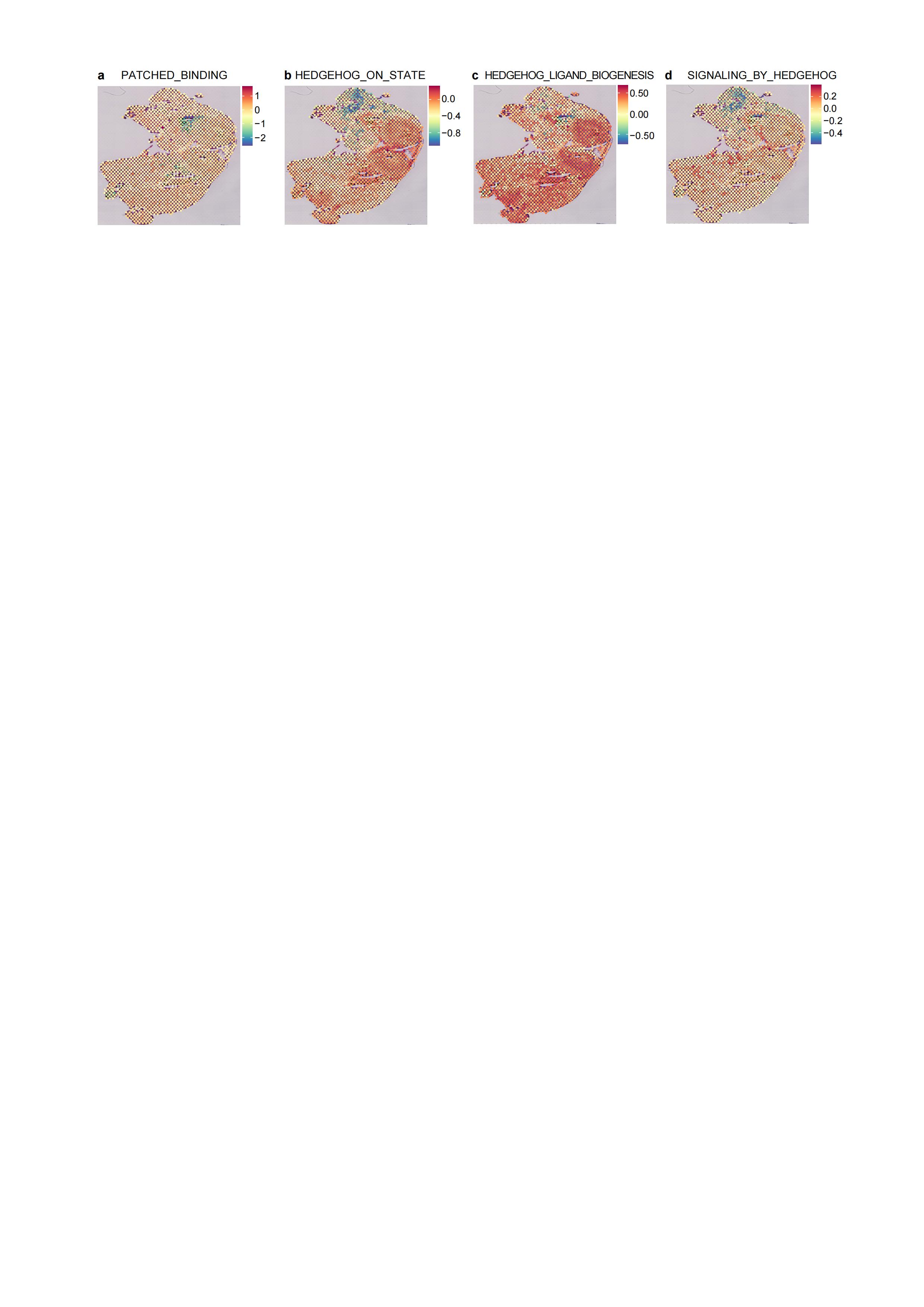
**Supplementary Figure 3.** Spatiotemporal expression profiling of Hedgehog pathway-associated gene ontology modules. (a-d) Expression patterns of representative gene ontology (GO) modules related to HH pathway in the P10 femur sample.

**
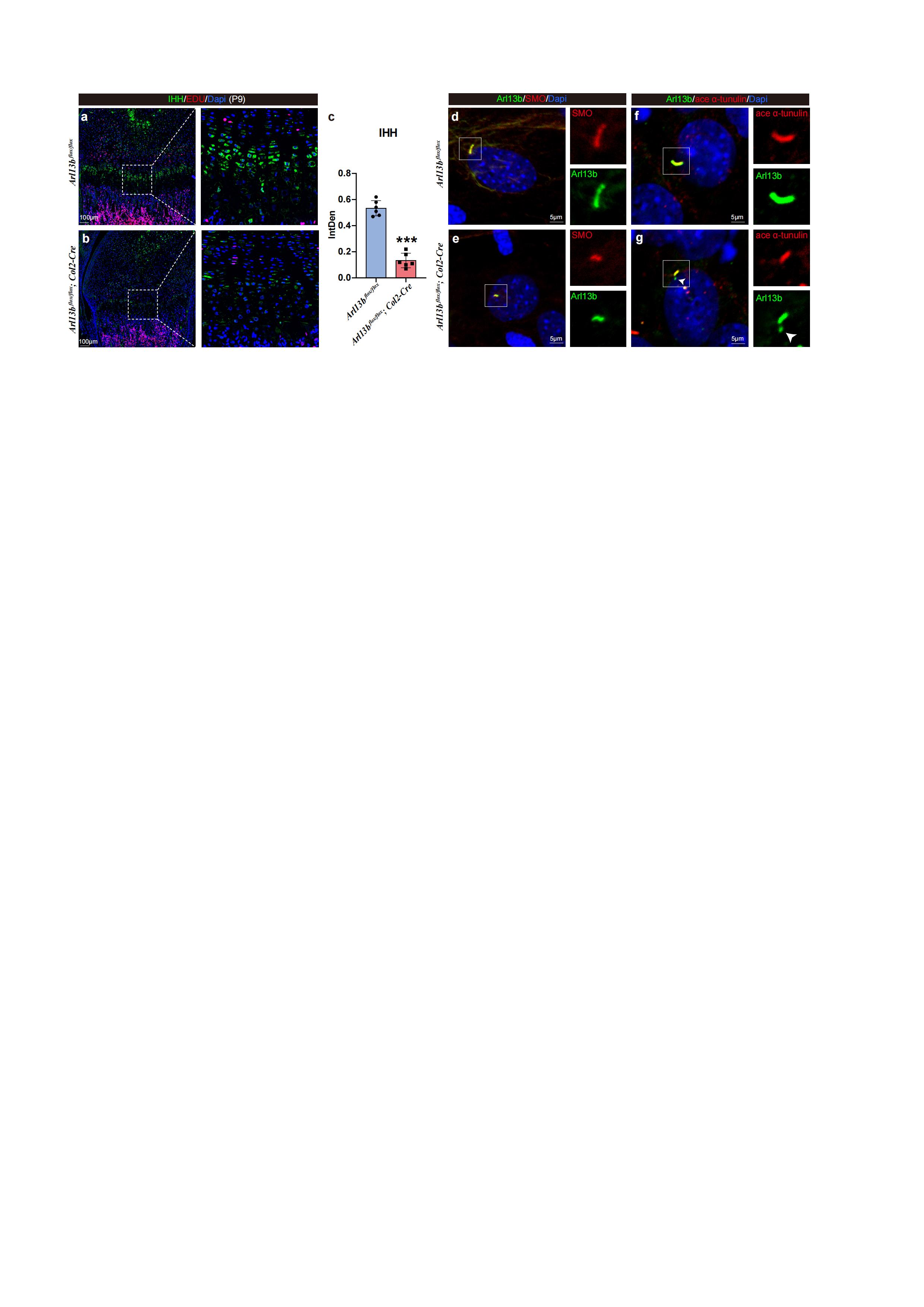
Supplementary Figure 4.** Inhibition of primary cilia formation leads to disrupted Hh signaling activation and cilia length stability in growth plate. (a & b) Immunofluorescence staining showing the Ihh^+^ cells distributed in the growth plate structures of the two group at P9. (c) The histogram showing the expression of Ihh in two groups at P9 (n=6). ***P < 0.001. (d & e) Immunofluorescence staining showing the colocalization of Smo in the primary cilia in both two groups. (f & g) Immunofluorescence staining showing the primary cilia structure in both two groups.

**
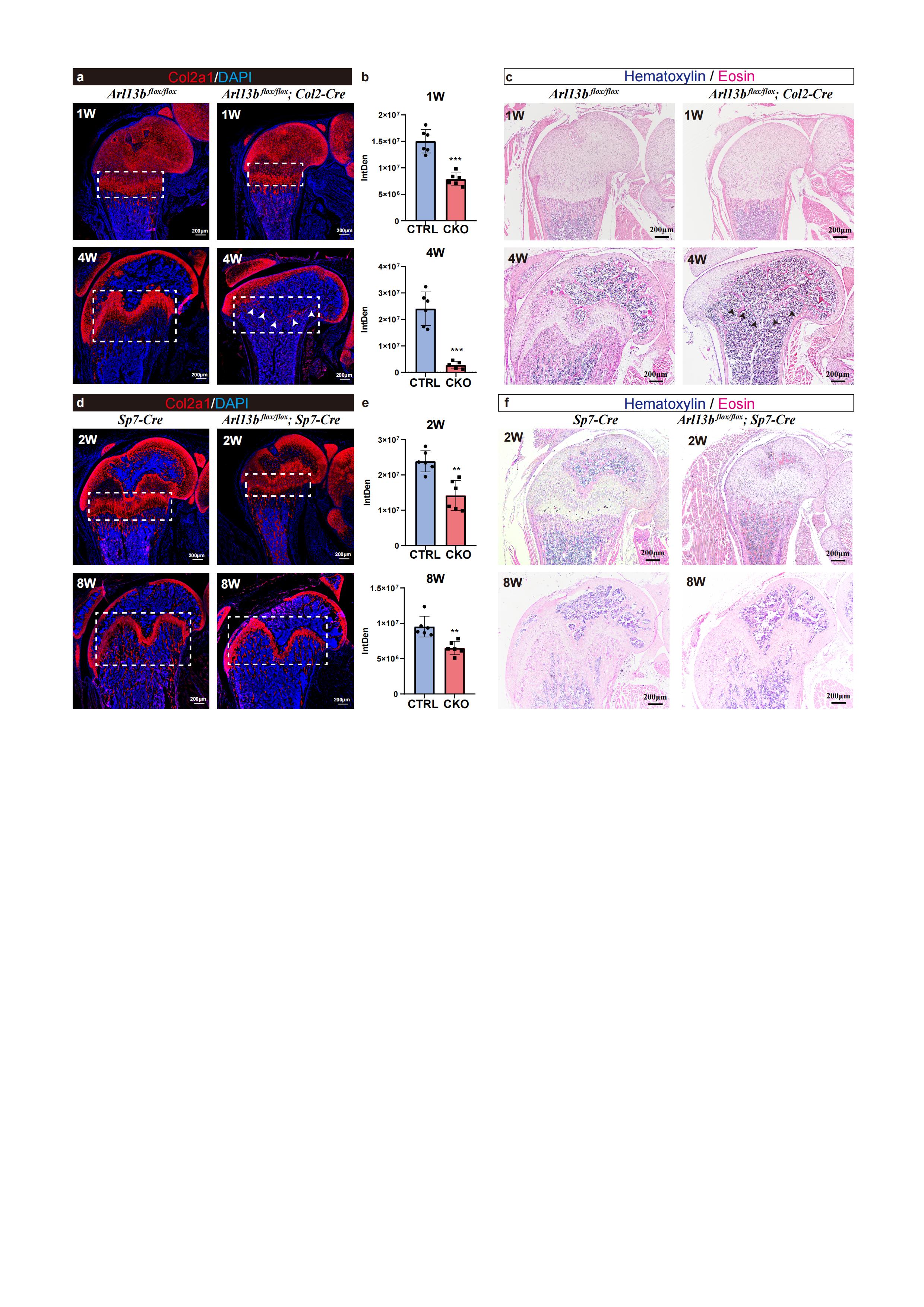
S****upplementary Figure 5.** Inhibition of primary cilia formation in growth plate chondroblasts and osteoblasts disrupts long bone development. (a-c) Immunofluorescent images and HE stainin of femur growth plate of *Arl13b ^flox/flox^* and *Arl13b ^flox/flox^; Col2-Cre* mice at 1 week and 4 weeks of age. The histogram showing the expression of Col2a1 in two groups at 1 week and 4 weeks of age (b). (d-f) Immunofluorescent images and HE stainin of femur growth plate of *Sp7-Cre* and *Arl13b ^flox/flox^; Sp7-Cre* mice at 2 weeks and 8 weeks of age. The histogram showing the expression of Col2a1 in two groups at 2 weeks and 8 weeks of age (e).

**
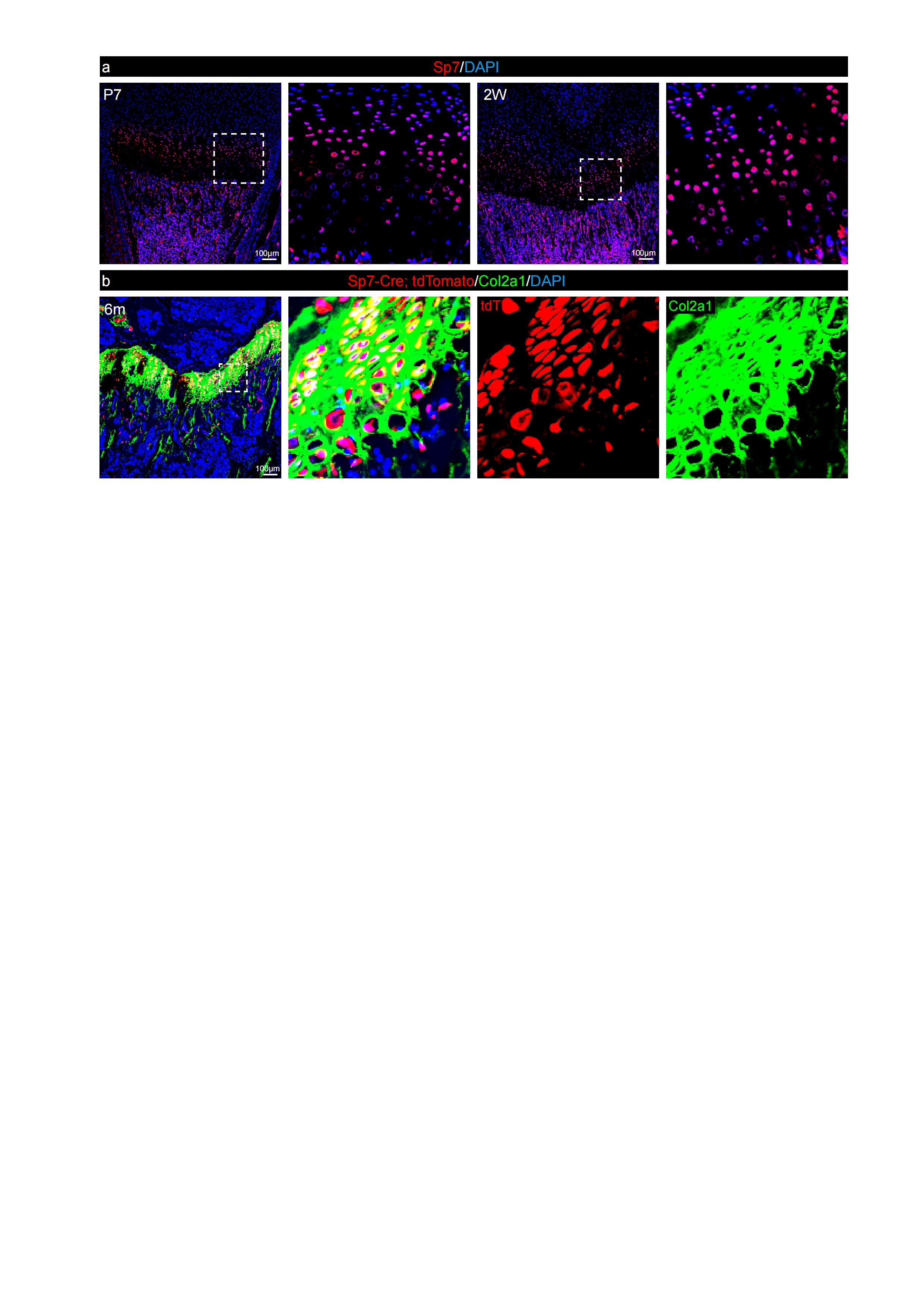
Supplementary Figure 6.** Distribution of Sp7^+^ cells within postnatal murine growth plate during long bone development. (a) Immunofluorescence staining showing the Sp7^+^ cells distributed in the growth plate structures of the wild type mice at P7 and 2-week-old. (b) Immunofluorescence staining showing the Sp7-lineage traced cells (tdTomato^+^) and Col2a1 staining within the growth plate structures of Sp7-Cre; tdTomato mice at 6-month-old.


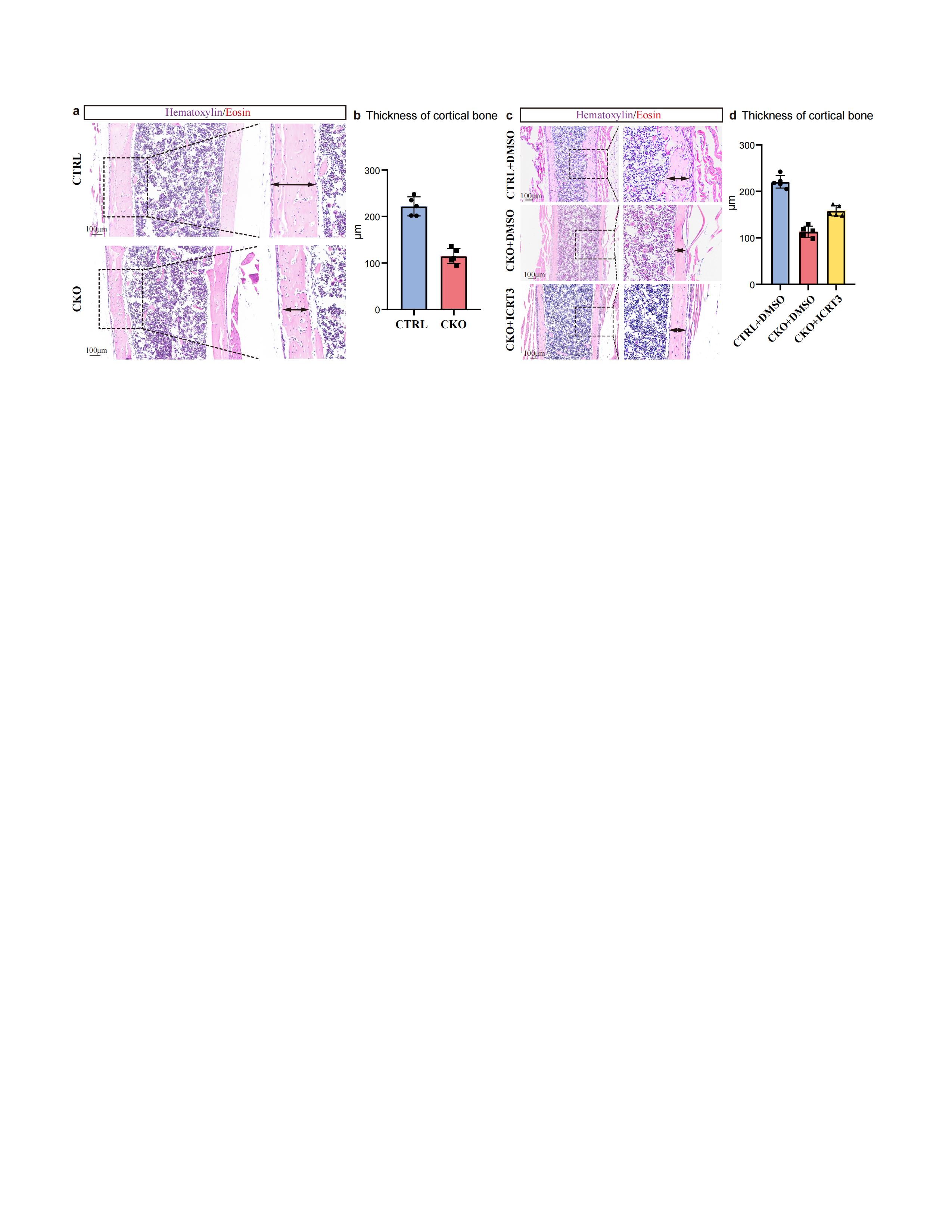
**Supplementary** **Figure 7**. Targeted inhibition of the Wnt signaling pathway partly rescued abnormal bone formation. (a) HE staining showing the cortical bone thickness of Sp7-Cre and Arl13b *^flox/flox^*; Sp7-Cre mice at 8 weeks of age. (b) The histogram showing the thickness of cortical bone between control and cKO mice (n=5) ***P < 0.001. (c) HE staining showing the cortical bone thickness among the three groups. (d) The histogram showing the thickness of cortical bone among the three groups (n=5) ns, ***P < 0.001.
